# Advances in immunotherapy for triple-negative breast cancer

**DOI:** 10.1186/s12943-023-01850-7

**Published:** 2023-09-02

**Authors:** Yang Liu, Yueting Hu, Jinqi Xue, Jingying Li, Jiang Yi, Jiawen Bu, Zhenyong Zhang, Peng Qiu, Xi Gu

**Affiliations:** 1 Department of Oncology, Shengjing Hospital of China Medical University, Shenyang, 110004 Liaoning Province China; 2 Department of Health Management, Shengjing Hospital of China Medical University, Shenyang, 110004 Liaoning Province China; 3 Department of Anesthesiology, Shengjing Hospital of China Medical University, Shenyang, 110004 Liaoning Province China

**Keywords:** Triple-negative breast cancer, Immunotherapy, Immune checkpoint, Drug resistance, Tumor microenvironment

## Abstract

**Background:**

Immunotherapy has recently emerged as a treatment strategy which stimulates the human immune system to kill tumor cells. Tumor immunotherapy is based on immune editing, which enhances the antigenicity of tumor cells and increases the tumoricidal effect of immune cells. It also suppresses immunosuppressive molecules, activates or restores immune system function, enhances anti-tumor immune responses, and inhibits the growth f tumor cell. This offers the possibility of reducing mortality in triple-negative breast cancer (TNBC).

**Main body:**

Immunotherapy approaches for TNBC have been diversified in recent years, with breakthroughs in the treatment of this entity. Research on immune checkpoint inhibitors (ICIs) has made it possible to identify different molecular subtypes and formulate individualized immunotherapy schedules. This review highlights the unique tumor microenvironment of TNBC and integrates and analyzes the advances in ICI therapy. It also discusses strategies for the combination of ICIs with chemotherapy, radiation therapy, targeted therapy, and emerging treatment methods such as nanotechnology, ribonucleic acid vaccines, and gene therapy. Currently, numerous ongoing or completed clinical trials are exploring the utilization of immunotherapy in conjunction with existing treatment modalities for TNBC. The objective of these investigations is to assess the effectiveness of various combined immunotherapy approaches and determine the most effective treatment regimens for patients with TNBC.

**Conclusion:**

This review provides insights into the approaches used to overcome drug resistance in immunotherapy, and explores the directions of immunotherapy development in the treatment of TNBC.

## Introduction

Breast cancer has become the commonest malignancy among women all over the world, and is related to the highest mortality rates from the disease. Triple-negative breast cancer (TNBC) is an entity that tests negative for the expression of the estrogen receptor (ER), progesterone receptor (PR), and human epidermal growth factor receptor 2 (Her-2), it is characterized by low differentiation, high invasiveness, a propensity for local and distant metastases, poor prognosis, and high recurrence rates [1]. Gene expression analysis shows that immune markers, mesenchymal phenotypes, androgen receptors, stem cell markers and basic markers are all associated with TNBC [2]. Based on findings from transcriptome research, TNBC may be divided into six subtypes, namely, basal-like 1, basal-like 2, mesenchymal stem cell, immunomodulatory, mesenchymal, and luminal androgen receptor [3]. In addition to the extracellular matrix (ECM), numerous immune cells in the tumor microenvironment (TME), including tumor-infiltrating lymphocytes (TILs), antigen-presenting cells, and fibroblasts contribute to the progression and metastasis in TNBC. At present, immunotherapy has become a new choice for many refractory solid tumors. Immune checkpoint inhibitors (ICIs) include inhibitors of programmed death receptor-1 (PD-1), programmed death receptor-ligand 1 (PD-L1), and cytotoxic T-lymphocyte-associated antigen 4 (CTLA-4), which is the most well-known immunotherapeutic agents. Breast cancer has long been considered a “cold” tumor due to its limited T-cell infiltration and low tumor mutation burden. Nevertheless, TNBC exhibits a greater presence of infiltrating lymphocytes, thereby establishing a favorable immune microenvironment for the potential utilization of ICIs. TNBC also demonstrates a relatively substantial tumor mutation load, thereby offering an antigenic foundation for immune cell recognition. The expression of PD-L1 is notably elevated in TNBC, thereby presenting a promising target for the implementation of ICIs [4]. Immunotherapy holds significant clinical relevance for TNBC, as the combination of ICIs with chemotherapy has yielded promising therapeutic outcomes in TNBC patients. Subsequent investigations should be conducted to elucidate efficacious immunotherapy regimens, thereby offering novel approaches to enhance the therapeutic efficacy of TNBC.

## TME and therapeutic resistance in TNBC

**Fig. 1:**
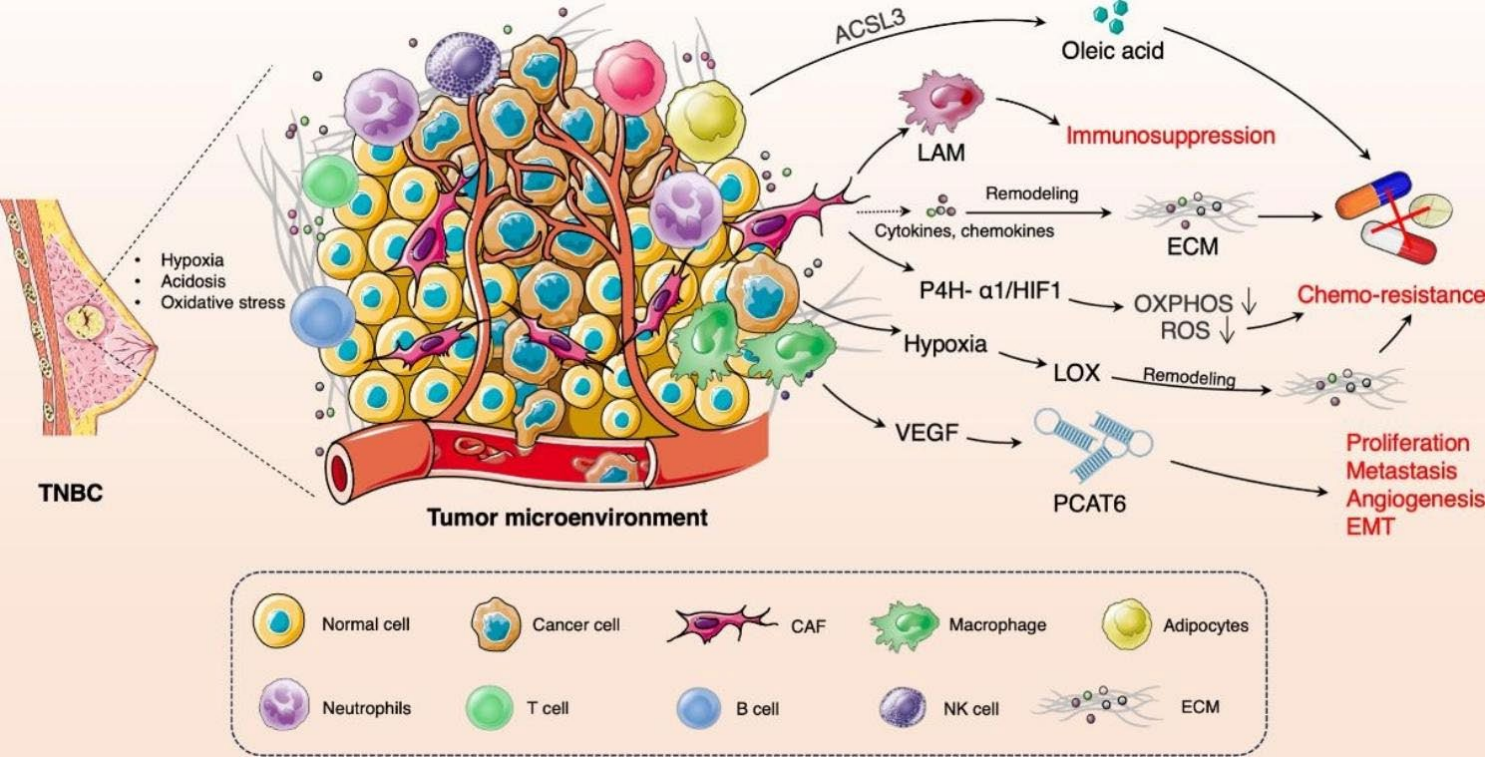
Interactions between components of TME induce therapeutic resistance in TNBC. The hypoxia of TME induces the activation of LOX and reshapes the ECM, leading to the formation of chemotherapy resistance. The activation of the P4H-α1/HIF-1 axis has been found to enhance the stemness of TNBC cells, resulting in a reduction in OXPHOS and ROS. CAF-induced lipid-associated macrophages induce immunosuppression of TNBC. The adipocytes induce the production of oleic acid to result in the protection of TNBC cells from ferroptosis by ACSL3. The M2 macrophages secrete VEGF and activate PCAT6 to promote the proliferation and metastasis of tumor cells through the modulation of VEFGR2

The tumor microenvironment (TME) encompasses tumor cells, cancer-associated fibroblasts (CAFs), immune cells including B and T lymphocytes, natural killer (NK) cells, tumor-associated macrophages (TAMs), the vasculature, and extracellular components including ECM, cytokines, chemokines, metabolites, and exosomes. The heterogeneity of TME within different subtypes of TNBC is notably pronounced [5]. The constituents of TME possess the ability to interact with one another, thereby altering the internal environment of the tumor and ultimately facilitating the development of resistance in TNBC. Research has indicated that non-cancerous cells within the TME play an important part in the development of cancer by facilitating tumor survival, progression, metastasis, and therapeutic resistance. The interaction between tumor cells and stromal cells in the TME, which are also implicated in tumor growth and drug resistance [2]. In addition, the proliferation, the metabolic remodeling, and the inhibition of cell apoptosis in tumor cells of TNBC can lead to hypoxia, acidosis, and oxidative stress within TME [6, 7]. Hypoxia, along with oxidative stress and acidosis, can induce the activation of lysyl oxidase (LOX) and subsequently reshape the ECM, and ultimately leading to the formation of chemotherapy resistance [8]. The activation of the collagen prolyl 4-hydroxylase P4H-α1/HIF-1 axis has been found to enhance the stemness of TNBC cells, resulting in a reduction in oxidative phosphorylation (OXPHOS) and reactive oxygen species (ROS) levels [9]. CAFs play a crucial role in promoting cancer progression and resistance to treatment through the secretion of cytokines, chemokines and ECM remodeling factors [10, 11]. Furthermore, CAF-induced lipid-associated macrophages (LAM) have been identified as mediators of immune suppression in breast cancer, particularly in TNBC [12]. The presence of ACSL3 in TNBC has been observed to result in the protection of TNBC cells from ferroptosis induced by mammary adipocytes [13]. Qiong et al. discovered that M2 macrophages secrete VEGF and activate PCAT6, thereby promoting the growth and metastasis of the cancer cells through the modulation of VEFGR2 in TNBC [14]. Zhang et al. have divided TNBC into macrophage-enriched subtype (MES) and neutrophil-enriched subtype (NES), revealing distinct mechanisms of immunotherapy resistance among different myeloid cell subtypes of TNBC [15]. The interactions between components of TME induce therapeutic resistance in TNBC are shown in Fig. 1.

## Remodeling of the TME

**Fig. 2:**
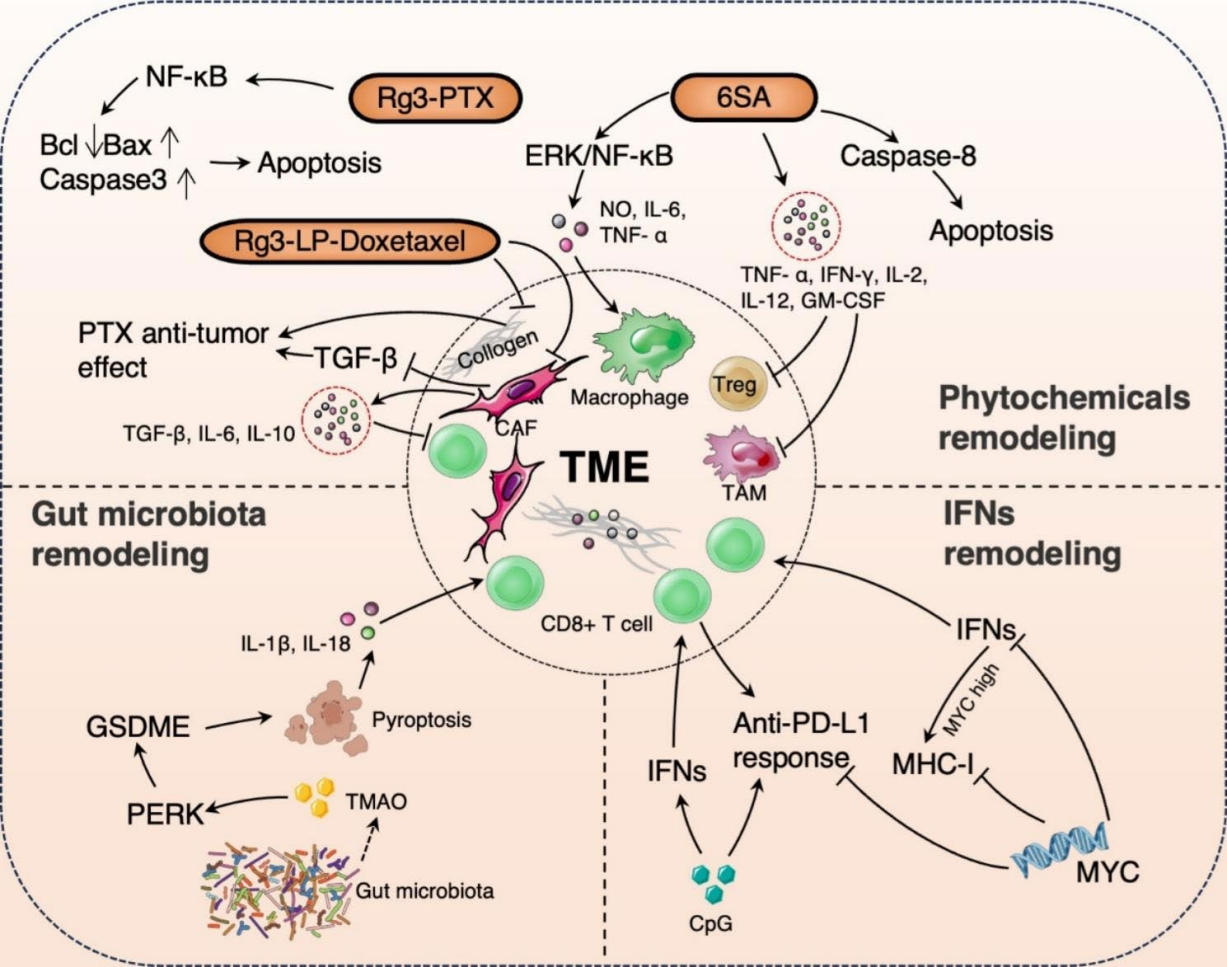
The diagram of remodeling of the TME in TNBC immunotherapy. Rg3-PTX-LPs induced apoptosis by NF-κB. Rg3-LP-Doxetaxel reduces CAFs, TGF-β, and collagen fibers in the TME to enhance the anti-tumor effect of Doxetaxel. 6SA triggers macrophages by MAPK as well as enhancing the secretion of NO and pro-inflammatory cytokine and inducing the induction of Caspase-8-mediated apoptosis, which activate macrophages and inhibit Treg and TAM. TMAO induces tumor cell pyrosis mediated by GSDME through activation of PERK, and releases IL-1β and IL-18, thereby enhancing the infiltration and killing function of CD8 + T cells. MYC protein expression is slightly downregulated after treatment with IFN-γ, IFNs may increase MHC-I expression in tumor cells that demonstrate high MYC expression. CpG-ODN offers synergistic effects with PD-1 inhibitors by producing IFN α and β as well as increasing CD8 + T cell infiltration in the TME

Abnormalities in the TME act as barriers to drug delivery, and are the main reasons for poor efficacy of chemotherapy and immunotherapy [16]. TNBC tissue exhibits severe fibrosis and abundant ECM deposition, which induces tumor vascular compression and reduces perfusion, which leads to poor drug delivery [17]. The Cytokines secreted by cancer-associated fibroblasts (CAFs) cause immunosuppression, tumor cell proliferation, epigenetic changes and ECM [18]. As TILs in the TME are closely related to the prognosis of TNBC, TME remodeling (recruiting CD4 + T cells, CD8 + T cells, and NK cells) improves the effect of immunotherapy which is a crucial approach used for treating breast cancer. The targets for reshaping the TME are therefore important for the treatment and prognosis of TNBC, including ameliorating tumor hypoxia, modulating tumor blood vessels, regulating CAFs and ECM, as well as influencing TAMs and dendritic cells (DCs). By either weakening the matrix barrier or enhancing the immunosuppressive microenvironment, the remodeling of TME can be achieved through the utilization of phytochemicals, targeting gut microbiota and metabolites ,and manipulating immunocytes and cytokines. These approaches collectively contribute to the optimization of TNBC anti-tumor therapy. (Fig. 2)

### The phytochemicals remodel TME

The chemotherapy drugs encapsulated in cholesterol liposomes generally have a limited effect on TNBC. This is mainly due to the formation of an impenetrable physical barrier by CAFs and their secreted products; these cells also secrete interleukin (IL)-10 and IL-6 and transforming growth factor-β (TGF-β), that collectively inhibit the expression of CD8 + T cells and have immunosuppressive effects within the TME [19, 20]. Notably, TGF-β signaling pathways inhibit CAFs, activate CD8 + T cells, enhance ECM degradation, increase blood perfusion and immune cell infiltration, and improve drug delivery, thereby improving the efficacy of immunochemotherapy [21, 22]. Ginsenoside Rg3, a component of ginseng, exhibits anti-tumor activity and inhibits growth and angiogenesis in human breast invasive ductal carcinoma xenografts in nude mice [23]. Rg3 has been used instead of cholesterol to develop a docetaxel-loaded Rg3 liposome, which can penetrate more deeply into the tumor, inhibit the CAFs ,TGF-β, and collagens to offer better chemosensitization in TNBC [24]. The combination of ginsenoside Rg3 with paclitaxel (PTX) can inhibit the nuclear factor-κB (NF-κB) pathway to enhance the cytotoxicity of chemotherapeutic agents in TNBC [25]. The liposomes based on Rg3 offer excellent encapsulation efficiency and drug-loading ability and can achieve tumor targeting and TME remodeling without any synthetic processes. They offer good clinical applicability and are expected to provide higher drug delivery efficiency for the treatment of TNBC.

Anacardic acid(6-Pentadecyl Salicylic Acid, 6SA) is found in the bark of dipteran plants and elicits the activation of macrophages by augmenting the MAPK signaling pathway, particularly ERK and NF-κB, as well as enhancing the release of nitric oxide (NO) and cytokines [26], which facilitates the activation of immune cells. 6SA reduces tumor volume without diminishing the number of TILs by inducing caspase-8 mediated cell apoptosis, and also concurrently augmenting the secretion of interferon-gamma (IFN-γ) and tumor necrosis factor-alpha (TNF-α), and improves the immune microenvironment of TNBC [26]. 6SA exhibits potent anti-tumor effects on TNBC in vivo with intact immune function, while also mitigating the myelosuppressive and leukopenic effects induced by paclitaxel [27]. Additionally, it enhances the tumor immune microenvironment in TNBC.

### Gut microbiota activate anti-tumor immunity

The response to ICIs is enhanced by the modulation of innate immunity, adaptive immunity, and the immunogenicity of tumor cells by gut microbiota. Specifically, the gut microbiota can influence the activity of DCs, mononuclear macrophages, and NK cells in terms of innate immune regulation. Additionally, the role of CD8 + T cells and CD4 + T cells could be influenced by the gut microbiota in terms of adaptive immune regulation. The metabolites, which are small molecules capable of disseminating from the intestinal primary site, promote the efficacy of ICI by influencing local and systemic anti-tumor immune responses [28]. Certain studies have identified subtypes of immunomodulatory organisms that induce a relatively active immune microenvironment in TNBC. The immunomodulatory (IM) subtype constitutes approximately 24% of TNBC cases and exhibits a more favorable prognosis in comparison to other subtypes. This particular subtype is more inclined to derive benefits from immunotherapy interventions, which involve the enhancement of immune signaling pathways and the augmentation of TILs. It has been found that the enrichment of *Clostridium* species and related metabolite trimethylamine oxide (TMAO) increases in tumor tissues of the IM subtype. TMAO can activate anti-tumor immunity and higher plasma TMAO levels are associated with better immune response. TMAO can induce tumor cell pyrosis mediated by GSDME protein through activation of endoplasmic reticulum kinase PERK, and secret inflammatory factors (IL-18 and IL-1β) into the TME, thereby enhancing the infiltration and cytotoxicity of CD8 + T cells. TMAO levels correlate positively with the amount of CD8 + T cells in the TME. In this context, TNBC patients with high CD8 + T cell counts demonstrate favorable IFN responses and higher immune cell infiltration [29]. In a clinical trial, choline (a precursor of TMAO) was employed to improve the efficacy of immunotherapy; this provided a new strategy for precision immunotherapy in TNBC. More metabolites of the gut microbiota have been shown to reshape the TME by regulating immune cells and affecting the efficacy of cancer immunotherapy, including ICIs. Therapeutic strategies for intestinal microbiome combined with ICI include FMT, probiotics, prebiotics, engineering bacteria, bacteriophage, and other strategies to enhance ICI response [30]. However, little is known regarding the potential molecular mechanisms related to gut microbiota and the impact of their metabolites on the efficacy of ICI in TNBC. Future studies are therefore needed to further explore the efficacy of microbiota in the treatment of TNBC, as this may help improve patient prognosis.

### IFNs activate anti-tumor immunity

The involvement of IFNs in the immunotherapy response against tumors presents a dual nature. Initially, IFNs can stimulate dendritic cells, thereby facilitating the cross-activation of CD8 + T cells specific to the tumor. However, prolonged exposure to IFNs can elicit negative feedback mechanisms, which faciliates the depletion of T cells and the induction of immunosuppressive effects. By modulating the immune response of IFNs, it is possible to augment the body’s immune response to tumors [31]. Studies have demonstrated that IFN signaling of tumor cells can restrict immune responses, whereas IFN signaling of adaptive and innate immune cells can enhance the immune response [32]. IFN-α and IFN-β, known as Type I IFN, have a wide range of impacts on different immune cells. such as augmenting the cytotoxic potential of NK cells and their ability to secrete IFN-γ, as well as facilitating the differentiation, maturation, and migration of antigen-presenting cells. On the other hand, IFN-γ, classified as a type II IFN, possesses antiviral, anti-tumor, and immunomodulatory properties [33], which are capable of activating other immune cells and fostering the immune response. Previous studies have found that MYC is overexpressed in some cases of TNBC and it promotes immunosuppression by inhibiting the IFN signaling pathway [34]. The MYC signaling pathway is related to low PD-L1 expression in cancer cells. Some studies suggest that patients with TNBC who demonstrate high MYC expression have lower major histocompatibility complex class I (MHC-I) expression [35]. MYC inactivation may therefore restore MHC-I expression, CD8 + T cell infiltration, and the anti-PD-L1 response. As MYC protein expression is slightly downregulated after treatment with IFN-γ, IFNs may increase MHC-I expression in tumor cells that demonstrate high MYC expression.

Unmethylated cytosine-phosphorothioate-guanine (CpG) oligodeoxynucleotides are synthetic toll-like receptor 9 (TLR9) agonists that stimulate plasmacytoid dendritic cells (DCs) to produce IFN α and β. They also activate T and B cells, recruit NK cells, induce humoral and cellular immunity, and upregulate IFN release; these promote the infiltration of antigen-specific CD8 + T cells [36]. Based on their chemical composition and in-vitro activity, CpG-oligodeoxynucleotides are mainly divided into three categories: A, B, and C. Studies have found that low doses of CpG-B can significantly inhibit tumor growth and offer synergistic effects with PD-1 inhibitors. Although higher doses of CpG-C may achieve the same anti-tumor effect as CpG-B, CpG-C offers more efficacy than CpG-B when combined with anti-PD-1 inhibitors [37]. The challenge of TNBC treatment interventions lies in effectively targeting IFNs to reshape the immune microenvironment, to guide rational combination therapy.

## Targeting immunotherapy resistance in TNBC

**Fig. 3:**
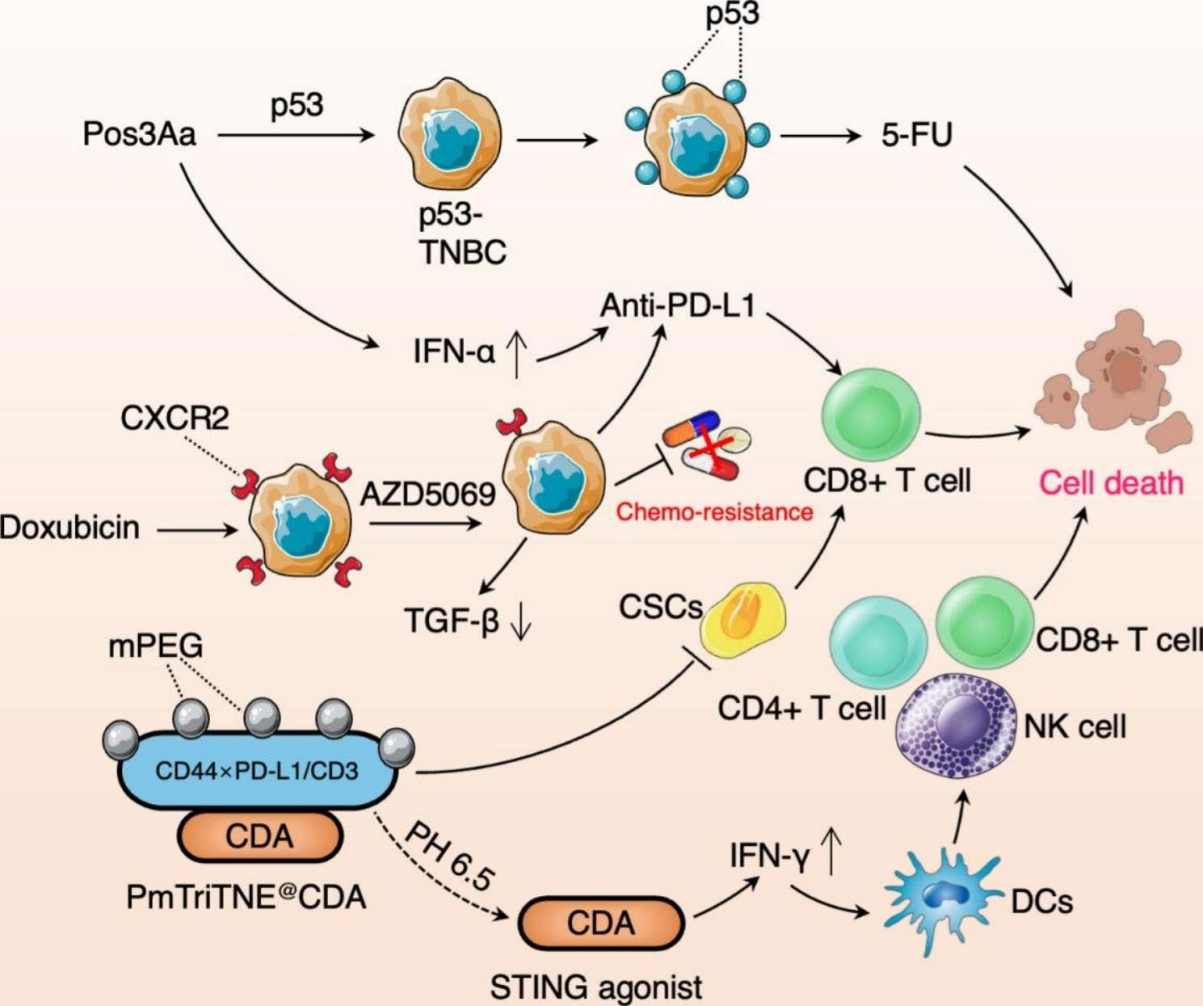
The diagram of overcoming TNBC Immunotherapy Resistance. Pos3Aa-p53 also significantly enhances the sensitivity of p53- deficient TNBC cells to the chemotherapy drug, 5-fluorouracil, and increases IFN. The combination of the CXCR2 antagonist, AZD5069, with doxorubicin, has been found to inhibit doxorubicin-mediated CXCR2 overexpression and reduce chemoresistance and TGF-β expression in TNBC. PmTriTNE@CDA releases STING agonist CDA to upregulate the expression of IFN-γ, promote the maturation of DCs, and recruit CD4 + T cells, CD8 + T cells, and NK cells. CSCs are suppressed by PmTriTNE@CDA to recruit CD8 + T cells

Although ICI is effective in some TNBC patients who experience a prolonged response, certain patients either have no response or acquire drug resistance [38]. Current understanding of the mechanisms of acquired resistance to ICIs is considerably limited; this inhibits the effective development of next-generation immunotherapy. Relevant studies on immunotherapy resistance have been described in Fig. 3.

### p53 delivery combined with PD-1 inhibitors

Numerous researches have shown that treatment with PD-1/PD-L1 inhibitors is more effective in cancers with more CD8 + T cells which may be used as predictive and therapeutic biomarkers for anti-PD-1 therapy [39, 40]. Notably, certain advanced TNBC cases lacking CD8 + infiltrating T cells demonstrate resistance to PD-1/PD-L1 inhibitors. The mutation frequency of tumor suppressor protein p53 may reach up to 80% in TNBC [41]; this may therefore be used as a potential biomarker. As the deletion or mutation of p53 can promote immune escape of cancer cells, restoring P53 activity in deficient tumors may potentially overcome resistance to PD-1/PD-L1 therapy. In this context, the naturally formed Pos3Aa protein crystal in *Bacillus thuringiensis* can be used as a carrier. Protein crystal-mediated p53 delivery can restore function in p53 deficient cancer cells, inducing significant apoptosis and cell cycle arrest. It is worth noting that Pos3Aa-p53 also significantly enhances the sensitivity of p53- deficient TNBC cells to the chemotherapy drug, 5-fluorouracil, and provides a new combined approach for the treatment of TNBC [42]. The strategy of Pos3Aa-p53 crystals combined with anti-PD-1 antibodies is safe, and significantly increases the number of IFNs, CD4+, CD44+, and CD8 + CD44 + memory T cells, thereby enhancing the curative effect to anti-PD-1 therapy [43]. In summary, these data confirm that intracellular p53 transmission may be an efficacious approach for improving the immunogenicity of p53 mutant tumors. The combination therapy of p53 restoration and ICIs in TNBC has considerable potential in overcoming resistance to ICIs. However, further studies are needed regarding the mechanisms involved; high-level evidence-based clinical trials are also needed for future clinical application.

### CXCR2 inhibitor combined with ICIs

CXCR2 chemokine receptor 2 (CXCR2) is mainly expressed by neutrophils. Notably, TNBC demonstrates higher levels of CXCR2 expression than other types of breast cancer [44]. In this context, CXCR2 participates in the treatment of drug resistance by maintaining of stemness of cancer stem cells (CSCs). The combination of the CXCR2 antagonist, AZD5069, with doxorubicin, has been found to inhibit doxorubicin-mediated CXCR2 overexpression and reduce chemoresistance and TGF-β expression in TNBC. An in vitro study found the combination of 30 nM of AZD5069 and 200 nM of atezolizumab to induce a notable cytotoxic effect (p = 0.0065); this indicates that in addition to the reduction of chemoresistance, CXCR2 inhibition enhances the efficacy of PD-L1 inhibitor [45]. Clinical trials and in vivo experiments are needed to evaluate CXCR2 inhibitor-based combination therapy in specific patient cohorts. This may provide breakthroughs and improve survival rates in TNBC patients.

### PmTriTNE@CDA nanotechnology

CD44 and PD-L1, which have overexpression in TNBC, play a important role in CSC stemness immune escape [1].In this context, a methoxy polyethylene glycol-covered CD44 × PD-L1/CD3 three specific T cell nano adapter (TriTNE) was loaded with c-di-AMP (CDA), the agonist of STING pathway, by using nanotechnology to develop PmTriTNE@CDA. Methoxy polyethylene glycol coverage blocks the antigen-binding of TriTNE; this dual targeting strategy (of CD44 and PD-L1) may improve selectivity and binding affinity to tumors. STING agonists can upregulate the expression of type I IFNs in TME, promote DCs maturation, activate T cells via tumor antigen presentation, and recruit CD8 + T cells and NK cells to the tumor lesion, thereby improving the immunogenicity of TNBC [46]. PmTriTNE@CDA targets CD3 and PD-L1 while inherently activating IFN signals; CSCs are suppressed and eliminated, and they interact synergistically with CDA to overcome the heterogeneity of TNBC and immunotherapeutic resistance.

## ICI monotherapy

ICIs eliminate cancer cells by releasing the “braking” mechanism of the immune system against cancer-attacking molecules. The two most prominent ICIs block CTLA-4 (CD152) and target PD-1 (CD279) and PD-L1 (CD274 or B7 homologue 1) [47]. A large-scale multi-omics analysis demonstrated the unique microenvironment characteristics and immune escape mechanism of TNBC. The types of microenvironment included the following: (1) immune desert type: unable to attract immune cells (related to MYC gene amplification), (2) immunosuppression type: chemotaxis present, but innate immunity is inactivated and tumor antigen levels are low, possibly contributing to immune escape (possibly linked to mutations in the phosphoinositide-3-kinase (PI3K)-AKT pathway), and (3) immunoinflammatory type: demonstrating high expression of immune checkpoint molecules. The results of this study are helpful for individualizing immunotherapy in TNBC patients. Notably, ICIs may be effective for the immunoinflammatory type and may transform the TME from an “immune cold” to an “immune hot” state in the immune desert and immunesuppression types.

### Anti-PD-1/PD-L1 therapy

PD-1 is an important immunosuppressive molecule that is mainly found on immune cells [48] and is typically expressed on the surface of T, B, NK, and myeloid cells. PD-L1 may be expressed by tumor and activated T cells, macrophages, and CAFs [49]. The binding of PD-1 to its ligand PD-L1 trigger SH2 protein tyrosine phosphatase 2 (SHP2), this downregulates the PI3K/AKT axis, inhibits the proliferation of T and B cells in peripheral tissue, induces programmed T cell death, and prevents autoimmune diseases, thereby maintaining immune homeostasis. However, this also provides the possibility of immune escape in tumor cells [50]. The Food and Drug Administration (FDA) of United States has approved the following PD-1/PD-L1 inhibitors: nivolumab, pembrolizumab, atezolizumab, cemiplimab, avelumab, and durvalumab [51]. Certain studies have reported that PD-L1 is present in around 20–30% of cases of TNBC, and is associated with lymphocyte infiltration and histological grades. In combination with extracellular signal-regulated kinase 1/2 inhibitors, PD-1/PD-L1 inhibitors show greater effects in TNBC cell lines than in non-TNBC cells [52]. Phase I clinical trial KEYNOTE-012 showed that pembrolizumab has good safety and object response rate (ORR) in TNBC patients [53]. Studies have confirmed that monotherapy of pembrolizumab offers sustained anti-tumor activity in patients with early and advanced PD-L1-positive TNBC (with a combined positive score [CPS] of ≥ 1) [54–56]. In phase I clinical trial (NCT02838823), PD-1 inhibitor JS001 shows good safety and effectiveness in metastatic TNBC patients who failed in multi-line treatment [57]. In the Phase I clinical trial (NCT01375842), the monotherapy of PD-L1 inhibitor atezolizumab provides long-lasting clinical benefits in mTNBC patients [58]. Phase I JAVELIN study showed that Avelumab, a PD-L1 inhibitor, had an ORR of 44.4% and 2.6% in 58 TNBC patients with PD-L1 ≥ 10% and < 10%, respectively [59].

However, the monotheraputic efficacy of PD-1/PD-L1 inhibitors is limited in TNBC and influenced by numerous factors. The resistance of breast cancer to ICIs also hinders their clinical application.

### Anti-CTLA-4 therapy

The CD28 and CTLA-4 receptors on T cells bind to CD80/CD86 to provide positive and negative feedback, respectively, for T cell activation. CTLA-4 is considered to be a necessary suppressor of the adaptive immune response, and is capable of maintaining peripheral tolerance, binding to CD80/CD86 on activated T cells, competitively inhibiting CD28, and binding to B7 co-stimulatory molecules expressed on antigen-presenting or tumor cells; this reduces the binding of B7 to CD28 and downregulates the expression of T cells [60]. CTLA-4 hinders the growth of T cells, progression of the cell cycle, production of IL-2, and differentiation of T cells. It is therefore a specific regulator of the CD4 + T cell response, and thereby maintains T cell homeostasis [61]. Some studies have analyzed the CTLA-4 expression among different types of breast cancer in The Cancer Genome Atlas (TCGA), it is demonstrated that the expression of CTLA-4 is found to be the highest in TNBC. Notably, it may be controlled by hsa-mir-92a to form a competing endogenous ribonucleic acid (RNA) network, which affects the prognosis of TNBC patients via the leukocyte and T cell activation pathways [61]. In this context, CTLA-4 expression is negatively associated with the T-cell activation score and survival rate (P = 0.0061) [62]. Notably, a study found CTLA-4 levels to be significantly higher in tissue samples from lymph node metastases than in those from the primary breast tumor. Additionally, the patients with high expression of CTLA-4 demonstrated a significantly increasing in axillary lymph node metastases [63]. CTLA-4 blocking may therefore activate or restore T cell function. In addition to inhibiting axillary lymph node metastasis, it can also enhance immune defense mechanisms and tumoricidal effects in breast cancer. In a study, combined preoperative tumor cryoablation and ipilimumab therapy demonstrated potential intratumoral and systemic immune effects with better safety; this suggests the possibility of a synergistic anti-TNBC immunity effect [64]. None of the CTLA-4 inhibitors has been currently approved for sole use in TNBC. However, combined pembrolizumab and chemotherapy with subsequent pembrolizumab monotherapy has been used for the adjuvant treatment of early high-risk TNBC with a PD-L1 CPS of ≥ 20.

### Combination of two ICIs

Both PD-1 and CTLA-4 are highly expressed in regulatory T cells (Treg). In this context, PD-1 can promote Treg cell proliferation in the presence of ligands. Numerous tumors demonstrate high infiltration by Treg cells (including CD8 + and NK cells), which inhibit the immune response. Blocking the PD-1 pathway may therefore enhance the immune response by reducing the inhibitory activity of Treg cells [65]. As CTLA-4 and PD-1 prohibit T cell function via different mechanisms, it may be reasonable to use CTLA-4 and PD-1 inhibitors to treat TNBC. Some studies have shown that the combination of two types of ICIs or ICIs with chemotherapy drugs offers a better response rate than monotherapy with either agent [66, 67]. However, 55% of grade 3 or 4 side effects occur in patients receiving ipilimumab and nivolumab; the corresponding incidence is 16.3% and 27.3% in those who receive nivolumab and ipilimumab, respectively. Notably, dual blockade with durvalumab (anti-PD-L1 monoclonal antibody) and tremelimumab (anti-CTLA-4 monoclonal antibody) activates and effectively expands T cell populations. PD-L1 and CTLA4 inhibitors may influence the crosstalk between B and T cells, inducing plasma cells to produce specific antibodies in the TME. These findings suggest that patients with TNBC are prone to benefit from combined treatment with durvalumab and tremelimumab [66]. Although this study had an exploratory design and a small sample size for verification of reproducibility, it confirmed the feasibility and effectiveness of combining two ICIs to a certain extent. The findings may be validated by future preclinical or clinical studies including larger sample sizes.

## Combination of ICIs and other treatments

### ICIs combined with chemotherapy

A meta-analysis that included early-stage TNBC patients showed that the use of ICIs in combination with anthracyclines and taxanes significantly increased the pathological complete response rate (pCR); in addition, ICI-chemotherapy drug combinations offered a lower incidence of adverse events compared with platinum chemotherapy [68]. Metastatic TNBC (mTNBC) is usually treated using chemotherapy; however, the objective response rate (ORR) with single-agent chemotherapy is only 10 − 30% and that of multidrug combination therapy can reach 63% [69, 70]. Clinical trials have found that cisplatin and doxorubicin significantly increase T-cell infiltration in patients with TNBC who subsequently receive nivolumab. Based on the high response rate to anti-PD-1 treatment and the upregulation of immune-related genes, cisplatin or doxorubicin induction may be considered to initiate tumor responses to PD-1 therapy. Clinical data on TNBC indicate that these chemotherapy agents can induce the formation of a more favorable TME in the short term, thereby improving the efficacy of ICIs [71]. Several clinical trials show that multiple neoadjuvant chemotherapy combined with Pembrolizumab regimens showed a favorable PCR rate in the treatment of early TNBC [72, 73]. In a study on early-stage TNBC, the combination of chemotherapy with pembrolizumab in the neoadjuvant setting provided a higher pCR (64.8%) than that of placebo (51.2%) [74]. The combination of anthracycline and taxane chemotherapy with durvalumab as a new adjuvant treatment can improve the prognosis of early TNBC patients [75, 76]. In the phase III clinical trial NeoTRIPaPDL1, the addition of PD-L1 inhibitor atezolizumab to neoadjuvant chemotherapy haven’t significantly improved the pCR rate of TNBC patients compared to neoadjuvant chemotherapy alone [77]. In the IMpassion031 trial, which compared atezolizumab combined with albumin-bound PTX and anthracycline with placebo and chemotherapy, the pCR was higher in the atezolizumab group than in the placebo group (58% vs. 41%, respectively) [78].

In IMpassion130 trial, which compared the efficacy of atezolizumab combined with albumin-bound PTX (experimental group) with that of albumin-bound PTX monotherapy (control group), the experimental group exhibited a longer median progression-free survival (PFS) and the median overall survival (OS) compared to the control group. In PD-L1 positive patients, the median PFS was longer in the experimental group than in the control group [79]. The results showed that atezolizumab combined with albumin-bound PTX offered a better prognosis than single-drug therapy in advanced TNBC patients, especially in PD-L-positive patients. However, in Phase III clinical study IMpassion131, the combination of atezolizumab and paclitaxel in the treatment of metastatic TNBC did not significantly improved PFS in the PD-L1-positive population. In another trial that evaluated pembrolizumab with chemotherapy against placebo with chemotherapy for treating advanced TNBC, the subgroup with CPS of > 10 demonstrated a median OS of 23.0 months in the pembrolizumab group and 16.1 months in the placebo group. Chemotherapy combined with ICIs was, therefore, more effective than chemotherapy in patients with PD-L1-expressing (CPS ≥ 10) advanced TNBC [80]. The therapeutic effect and prognosis of immunotherapy may therefore be predicted before treatment in advanced TNBC patients (based on the expression of PD-L1). KEYNOTE-355 showed that the first-line treatment of pembrolizumab combined with chemotherapy significantly improved the OS for advanced TNBC with PD-L1 (CPS ≥ 10) [81].

An open-label, non-randomized, single-arm phase I/II clinical trial demonstrated favorable tolerability and efficacy of the combination therapy comprising eribulin and pembrolizumab for managing metastatic TNBC [82]. Similar clinical studies have achieved different results, such as in the IMpassion031 study and NeoTRIPaPDL1 study, which may be related to factors such as the pathological characteristics of the enrolled population, PD-L1 detection methods, and compatibility protocols.

### ICIs combined with radiotherapy

Despite significant advances in chemotherapy, endocrine therapy, and targeted therapy for breast cancer, radiotherapy continues to play an important role in TNBC. Radiotherapy recruits immune cells into the TME in various ways. It induces dying tumor cells to release danger signals. DCs combine with these danger signals to ingest antigens from cancer cells and transmit the antigens to lymph nodes. These are then presented to the initial T cells, thereby activating CD8 + and CD4 + T cells. This in turn triggers chemokine-induced recruitment of effector T cells to tumors [83]. Preclinical data indicate that radiotherapy combined with ICIs can not only enhance anti-tumor efficacy but also induce responses outside the radiation field. In a low immunogenicity mouse model of TNBC, radiotherapy upregulated the expression of genes containing immunogenic mutations. Vaccination with new epitopes encoded by these genes induced CD8 + and CD4 + T cells, thereby enhancing the immune response [84]. A study compared radiotherapy, anti-CTLA-4 antibody, and combined treatment with both in low- immunogenicity metastatic mouse breast cancer 4T1 cells; the findings suggested that combined treatment with ICIs and chemotherapy enhanced T cell infiltration, delayed growth of the primary irradiated tumor, improved survival rates, and inhibited lung metastasis [85].

In the TONIC trial, which had an adaptive design, mTNBC patients received nivolumab for 2 weeks and were then administered low-dose radiotherapy, the final ORR was 10%. The results indicate that CTLA-4 inhibitor therapy before low-dose radiotherapy offers better outcomes than other regimens, as the consumption of immunosuppressive Treg cells limits the function of CD8 effector cells and anti-tumor immunity. In this context, a multi-center phase 2 study evaluated the effectiveness and safety of combined radiotherapy and pembrolizumab in mTNBC patients. The study found that in the intention-to-treat cohort, the ORR of the unselected PD-L1 population was 17.6%; this was higher than that of mTNBC patients who had previously received ICI monotherapy [86]. However, the combination of immunotherapy and radiotherapy may be a double-edged sword. Owing to ICI-induced alterations in the balance between immune resistance and tolerance, increased tumor recognition is accompanied by higher immune response rates in normal tissues. On administration of radiotherapy, both tumor-specific and non-tumor-specific antigens may be released into the TME; this may activate autoreactive T cells, and possibly attack and damage normal tissues. Therefore, the combination of the two may increase the severity of adverse effects. Future large-scale clinical trials are therefore needed to further explore the best combination strategy for radiotherapy and immunotherapy.

### ICIs combined with gene therapy

There has been some progress in the administration of gene therapy with ICIs in TNBC, and studies are evaluating its immunotherapeutic effect (whether similar or superior). In the early stages, gene therapy utilized the oncolytic effect of Ad.sT, a protein created by fusing an adenovirus and a TGF β receptor II IgG Fc fragment (sTGFβ RIIFc). The Ad.sT interfered with TGF β-1 binding and inhibited TGF-β-dependent transcription when it was infected in MDA-MB-231 cells. More than 85% of the tumors of MDA-MB-231 xenografts in nude mice showed a reduction by the administration of Ad.sT [87]. Ad.sT could increase the amounts of CD4 + and CD8 + T lymphocytes in peripheral blood, thereby improving the immunotherapeutic effect of ICIs. Notably, a combination of PD-1 inhibitors and CTLA-4 inhibitors increases the efficacy of Ad.sT in inhibiting the metastasis and proliferation of tumor [88]. In this context, the LyP-1 receptor is highly expressed in breast cancer and three oncolytic viruses, namely, Ad.sT, AdLyp.sT, and mHAdLyp.sT; the latter two produce higher levels of the sTGFβRIIFc protein compared to viruses without LyP-1 modification. Intravenous injection of AdLyp.sT and mHAdLyp.sT has been found to induce a strong anti-tumor response in a bone metastasis model of TNBC, the delivery of AdLyp.sT in a 4T1 mouse model with normal immune function also enhanced the efficacy of anti- CTLA-4 and anti-PD-1treatment [89]. These findings suggest that it may be possible to develop more potential targeted immunotherapeutic drugs for TNBC.

In some studies, adenovirus-mediated herpes simplex virus(HSV)thymidine kinase was injected into mTNBC tumors; the injection site was then irradiated using stereotactic body radiotherapy (SBRT) and the patients subsequently received pembrolizumab. The median duration of treatment and OS in this study were 9.6 and 14.7 months, respectively. Notably, the median OS had more than doubled in patients who experienced clinical benefits; this indicated that the use of SBRT in conjunction with HSV thymidine kinase and gene therapy prior to pembrolizumab can improve the therapeutic efficacy of the latter in patients with mTNBC. This combined treatment regimen has also shown improved anti-tumor efficacy in a study on patients with liver metastases who had a low or negative PD-L1 status [90]. However, the patients in this study were not randomized, and the response rate to oncolytic viruses (alone or in combination with ICIs) did not exceed 12%. In addition, the combination of ICIs and SBRT only improved the response in preconditioned patients without liver metastasis (and demonstrated a shorter duration of response) [86]. Larger future randomized studies are therefore needed to evaluate the effect of each modality and its impact on the tumor microenvironment.

### ICIs combined with targeted therapy

#### ICIs combined with poly (adenosine diphosphate ribose) polymerase inhibitors

The enzyme, poly (adenosine diphosphate ribose) polymerase (PARP), plays a role in the repair of deoxyribonucleic acid (DNA) single-strand breaks. Notably, PARP inhibitors (PARPi) have been widely used in treating cancers with homologous recombination repair defects. In this context, TNBC is usually found in cases of familial breast cancer; approximately 50% of cases demonstrate BRCA1/2 mutations, which can disrupt DNA repair pathways [91]. Four PARPIs have been currently approved by the FDA of the United States, namely olaparib, rucaparib, niraparib, and talazoparib. Olaparib, and talazoparib monotherapy have been approved for advanced or metastatic human epidermal growth factor receptor 2-negative breast cancers with BRCA mutations [91–93]. Notably, PARPIs affect the TME by activating the IFN gene stimulating factor (STING) pathway in cancer cells. Certain studies have confirmed that in cases of BRCA1 deletion/mutation, PARPIs induce T cell recruitment, upregulate IFN responses through the cGAS-cGAMP-STING pathway, increase tumor neoantigens, upregulate PD-L1 expression, and increase tumor immunogenicity in TNBC cell lines.

In advanced or mTNBC, combined pembrolizumab and niraparib have demonstrated an ORR of 21% and a disease control rate of 49% [94]. In a cohort study where TNBC patients were treated with ICIs, combined avelumab and talazoparib offered an ORR of 18.2% [95]. The combination olaparib and durvalumab is safe in BRCA-mutated mTNBC [96]. It offered a high response rate in patients with homologous recombinant DNA repair pathway gene changes [97]. The mentioned studies have evaluated the potential synergistic effect of ICIs and PARPIs in the treatment of TNBC. The reports from other studies using combination therapy, including the NCT03101280 and NCT03544125, have not yet been published. Combined treatment needs to be based upon the identification of TNBC molecular subtypes (immune checkpoint positivity or negativity and high or low expression) and homologous recombinant defective gene types.

#### ICIs combined with anti-angiogenic drugs

Vascular endothelial growth factor (VEGF) is crucial in facilitating growth, survival, migration, invasion, angiogenesis, and increased vascular permeability in vascular endothelial cells. Studies have found that higher angiogenic enrichment scores are closely associated with higher age, lower TIL counts, and the immunomodulatory subtype of TNBC. Anti-VEGF therapy may therefore be used to promote immune checkpoint blockade. Notably, the immunostimulatory effects and toxicities of anti-VEGF drugs are highly dose-dependent. Low-dose anti-VEGF therapy can normalize blood vessels and improve anti-tumor immunity, while high doses can induce hypoxia and create an immunosuppressive TME [98]. Low doses of the anti-VEGF receptor (VEGFR)2 antibodies may induce more immune cell infiltration than regular doses; this provokes CD8 + T cells and macrophages to secrete osteopontin (OPN), a crucial bone matrix protein in cell movement, cytokine generation, and immune control [99]. OPN may promote the production of TGF-β, which upregulates the expression of PD-1 on immune cells. In patients with advanced TNBC, the combination of low-dose VEGFR inhibitors (apatinib and camrelizumab) has shown good tolerance and efficacy with a higher ORR than that of monotherapy. Notably, the approach of combining camrelizumab and VEGFR2 inhibitors has even shown good efficacy in patients with PD-L1 negative tumors or those receiving advanced lines of chemotherapy [100]. Certain studies have combined the VEGFR inhibitors famitinib and camrelizumab to treat patients with advanced TNBC. The combination provided an ORR of 81.3% and a median PFS of 13.6 months. All PD-L1 positive patients achieved an objective response, while only 69.2% of PD-L1 negative patients achieved an objective response (P = 0.030); this indicated that PD-L1 positive patients obtained more benefit from this protocol [101]. In addition, the PFS was higher in CD8 + patients than in CD8- patients; CD8- PD-L1- tumors showed the most unfavorable prognosis with an ORR of 60% and a median OS of 15.2 months. This suggests that immunohistochemistry for CD8 + and PD-L1 may be used for predicting the markers of clinical response. This study confirmed the effectiveness, safety, and feasibility of triple therapy in TNBC and demonstrated the potential of combined anti-VEGF, CD8+, and anti-PD-L1 therapy in the clinic. The prognosis of the combination may be evaluated in future studies.

#### ICIs combined with PI3K/AKT pathway inhibitors and chemotherapy

The PI3K/AKT/protein kinase B (PKB)/ mammalian target of rapamycin (mTOR) (referred to as the PAM pathway) regulates the proliferation, differentiation, apoptosis, and angiogenesis of tumor cells [102]. The activation of proto-oncogenes (*PIK3CA*, *AKT*, and *mTOR*) and inactivation of tumor suppressor genes (*PTEN*) are often observed in TNBC and are closely associated with proliferation and chemoresistance in these tumors [103]. An albumin nanoparticle containing PI3K-γ inhibitor eganelisib (IPI-549) and PTX enhanced the efficacy of α-PD1 with longer PFS and a better remission rate in TNBC [104]. The combination of Ipatasertib, an AKT inhibitor, with Atezolizumab + Paclitaxel/Nab-paclitaxel has demonstrated favorable efficacy in managing advanced and metastatic TNBC [105].

#### ICIs combined with 2-fluoro-L-focus (2 F-Fuc)

The B7 homologous 3 (B7H3) protein is a type I transmembrane protein. N-glycosylation of B7H3 induces immunosuppression in TNBC, indicating a poor prognosis in these patients; the glycosylated protein, therefore, represents a potential new target for immunotherapy. Compared with non-glycosylated B7H3, glycosylated B7H3 induces a reduction in total CD4 + T, CD8 + T, and NK cell counts in tumors and activates fewer cytotoxic CD8 + TILs; this suggests that glycosylated B7H3 interferes with the proliferation and activation of T cells. In this context, 2-fluoro-L-focus (2 F-Fuc) is an effective inhibitor of fucosylation, which enhances the activation of T cells by reducing B7H3 glycosylation and restoring the vulnerability of cancer cells to cytotoxic T-cell-mediated death. A study found combined treatment with 2 F-Fuc and anti-PD-L1 to significantly inhibit tumor growth in vivo [106]. Notably, 2 F-Fuc may enhance PD-L1 immunotherapy by blocking B7H3 core focusing, and combined treatment with PD-L1 may enhance the therapeutic effect in B7H3-positive TNBC. Targeting the FUT8-B7H3 axis may therefore represent an effective strategy for improving the therapeutic effect of ICIs in TNBC patients.

Furthermore, the efficacy of various pathway inhibitors, such as MEK1 inhibitor cobimetinib, HDAC inhibitor entinostat, ADC agents such as ladiratuzumab vedotin and trastuzumab deruxtecan in conjunction with ICIs, has been demonstrated to be promising in the treatment of TNB [107–110].

### ICIs combined with nanotechnology

Nanoscale materials have small particle sizes, stable properties, and high bioavailability. As a new area of interest in the field of cancer research, nano delivery systems are applied for drug delivery and early detection. Certain studies have shown that the combination of nanotechnology with immunotherapy ensures targeted delivery and stability of loaded drugs (via the application of nanocarriers) and enhances drug uptake and biocompatibility in breast cancer.

#### ICIs combined with recombinant nanoparticles

Nanoparticles carrying ozone (O3) were designed and synthesized in a laboratory and polylactic co-glycolic acid was used to wrap an O3-rich perfluoronaphthalane carrier; this was modified with iRGD or isotactic polypropylene oxide (iPPO) [111]. O3 is prepared by ionizing oxygen and blowing into an iPP (polyacrylic acid polymer lactic acid and glycolic acid perfluoronaphthalane) solution to form iPPO (O3-loaded iPP particles), a microwave (MW)-controlled release nanosystem. Notably, MWs have good penetration ability as electromagnetic waves [112], and iPP particles can stably carry O3 and store it in the carrier perfluoronaphthalane, which accumulates in tumor tissue after intravenous injection. After internalization of the nanoparticles into tumor cells, low-energy MW irradiation promotes the release and decomposition of O3 from perfluoronalkanes to produce ROS (especially highly toxic hydroxyl radicals), which induce tumor cell apoptosis and cell lysis without damaging normal tissues and organs [113]. The findings showed the combined treatment of iPPO and MW resulted in a 3.3-fold increase in the total apoptosis rate of 4T1 cells compared to the control group. The total apoptosis rate even reached 98.8% in MDA-MB-468 cells treated with iPPO, this proportion was higher than that of the control group (6.36%). Systemic use of anti-PD-1 inhibitors can inhibit the growth of primary and peripheral tumors to a certain extent. In this context, combining anti-PD-1 therapy with iPPO and MW offers inhibitory rates of 77.65% and 68.17% in primary and peripheral tumors, respectively. Anti-PD-1 therapy combined with iPPO and MW has shown the most effective tumor growth inhibition effect in all groups. It significantly improved CD4 + and CD8 + T cell infiltration in primary tumors and increased immunogenic cell death (ICD) and new antigens release; this effectively promoted the anti-tumor efficacy of PD-1 inhibitors. Notably, hydroxyl free radicals induce ICD, thereby triggering an anti-tumor immune response. Their binding to ICIs can promote the maturation of DCs, reverse immunosuppression in the TME, and enhance the systemic anti-tumor immune response [114].

Another approach for improving anti-PD-L1 therapy via ICD is the construction of a self-expanding biomimetic nanosystem (mEHGZ), created by glucose oxidase, wrapping epirubicin (EPI), and heme in zeolitic imidazolate framework-8 nanoparticles and coating them with calreticulin-overexpressing tumor cell membranes. EPI can induce ICD, glucose oxidase, and heme; this enhances ICD via cascade generation of ROS and promoting DC maturation and CTL infiltration [115]. Compared with the controls (such as EPI monotherapy), mEHGZ nanoparticles demonstrated the highest efficacy (86.0%) in inducing 4T1 cell apoptosis in a study. In combination with anti-PD-L1 therapy, mEHGZ effectively induced IFN-γ positivity. The CD8 + T cell tumor infiltration rate was 31.2%; this was 11.5-fold more than that observed in the EPI-treatment group. Compared with EPI-treated group, the tumor growth inhibition rate (82.02%) was also higher (36.41%). These results confirm that mEHGZ combined with anti PD-L1 therapy can induce a strong adaptive immune response in vivo. These two nanotechnology approaches enhance the sensitivity and efficacy of ICIs against TNBC via the PD1/PDL1 pathway and offer a promising approach for improving the response rate to ICIs.

#### ICIs combined with Mucin 1 messenger RNA nanovaccine

**Fig. 4:**
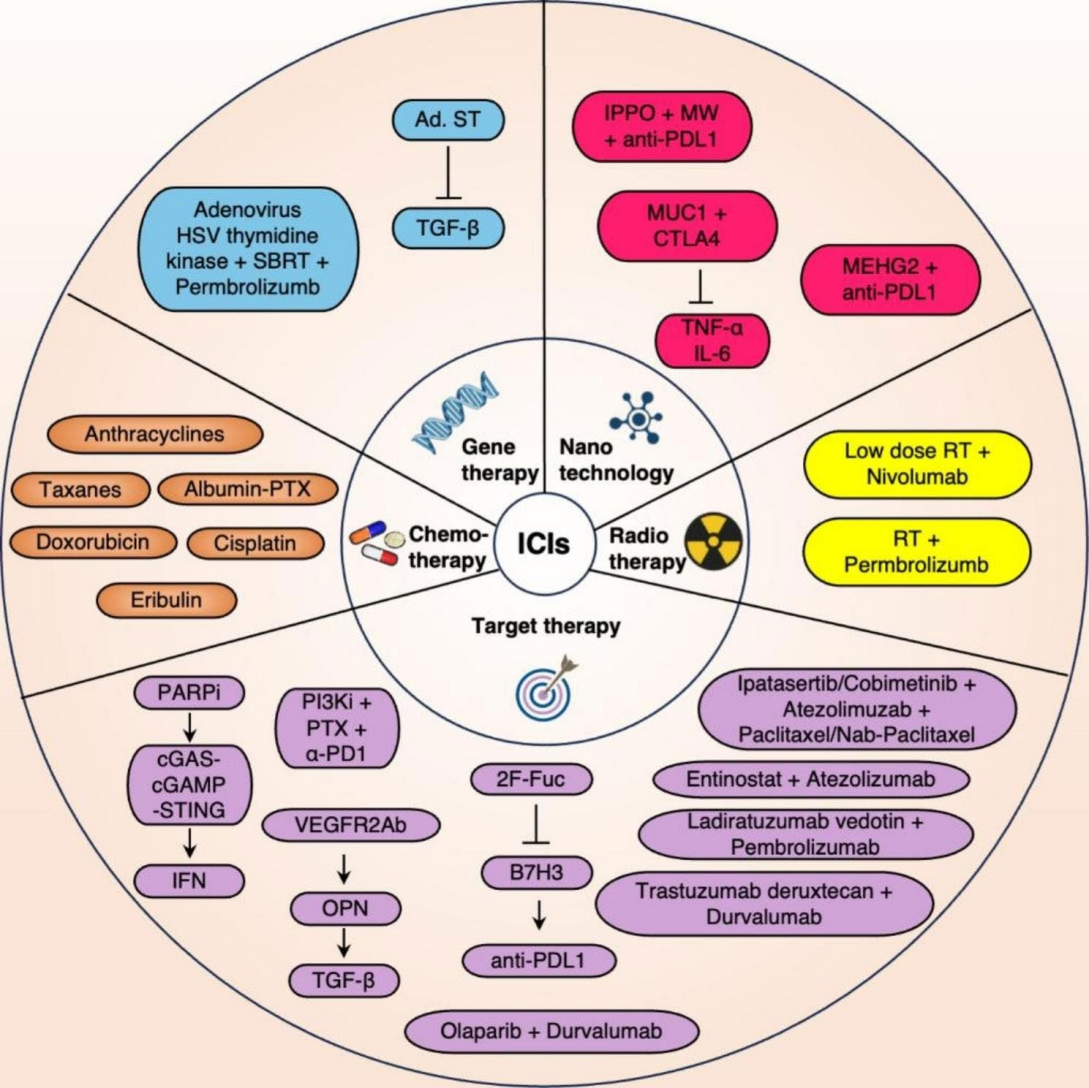
The diagram of combination of ICIs and other treatment. Anthracyclines, taxanes, cisplatin, doxorubicin and Albumin-PTX, and eribulin are used to combine with ICIs to enhance the clinical response in early and metastatic TNBC. Low-dose RT with Nivolumab and RT with Pembrolizumab enhance anti-tumor efficacy in TNBC. The Ad.sT interfered with TGF β-1 binding and improved the immunotherapeutic effect of ICIs. The regimen of adenovirus-mediated HSV thymidine kinase, SBRT, and pembrolizumab improve the therapeutic efficacy in patients with mTNBC, PARPIs upregulate IFN responses through the cGAS-cGAMP-STING pathway to upregulate PD-L1 expression. Low doses of VEGFR2 antibodies provoke CD8 + T cells and macrophages to secrete OPN and promote the production of TGF-β which upregulates the expression of PD-1. PI3K-γ inhibitor eganelisib (IPI-549) and PTX enhanced the efficacy of α-PD1 in TNBC. 2 F-Fuc enhances the activation of T cells by reducing B7H3 glycosylation and restoring the susceptibility of tumor cells to enhance PD-L1 efficacy. Olaparib, Ipatasertib, cobimetinib, entinostat, ladiratuzumab vedotin, and trastuzumab deruxtecan combined with ICIs and chemo drugs has demonstrated favorable efficacy in managing TNBC. The combining anti-PD-1 therapy with iPPO and MW offers an effective tumor growth inhibition effect. in a study. In combination with anti-PD-L1 therapy, mEHGZ effectively induced IFN-γ positivity in TNBC. The combination treatment with the CTLA-4 and MUC1 messenger RNA nanomachines is associated with lower expression of IL-6 and TNF-α, which may reshape the immunosuppressive TME

Mucin 1 (MUC1), a highly glycosylated tumor-associated antigen expressed on the external surface of epithelial cells [116], is a target in breast cancer immunotherapy. It binds to oxidized mannan and targets the mannose receptor on DCs to induce specific antibody and CD8 + T cell responses in breast cancer. In this context, a type of lipid/calcium/phosphate (LCP) nanoparticle was developed and used for creating a vaccine, which can effectively release the messenger RNA encoding MUC1 into the cytoplasm to activate tumor-specific T cells. In a study, the mice in the LCP-messenger RNA treatment group showed the highest MUC1-specific CTL-induced cytotoxicity; notably, immunization with the target MUC1 antigen improved CD8 + cytotoxicity. In another study on a 4T1 mouse model, LCP vaccines (with or without mRNA) were injected subcutaneously and anti-CTLA-4 antibodies were injected intraperitoneally. After multiple measurements of the tumor size within 25 days, the group receiving the MUC1 vaccine in combination with the CTLA-4 antibody demonstrated the smallest tumor size and the strongest anti-tumor activity (p < 0.001) as compared with the vaccine (p < 0.001) and anti CTLA-4 antibody (p < 0.01) groups. The findings support the potential of nanoparticle-based messenger RNA vaccines in combination with CTLA-4 inhibitors in immunotherapy for TNBC. Although the mechanism responsible for enhancing the efficacy of the combination is unclear, the nanoparticle delivery system improves the stability, sustainability, and expression level of messenger RNA-based vaccines [117]. Further studies on the injection of CTLA-4 inhibitors prior to vaccination with the MUC1 messenger RNA nanovaccine have shown that treatment with the combination is associated with lower expression of cytokines including IL-6, TGF-β, and TNF-α, and low Treg cell counts and bone marrow-derived inhibitory cells, this may reshape the immunosuppressive TME. The combination of ICIs and other treatments enhanced the antitumor effect in TNBC as shown in Fig. 4.

## Tumor vaccine

Tumor vaccines use cancer cells, antigens, or other related biological components to confer the host and produce a potent anti-tumor immune response. The vaccines may be used to treat and prevent the progression of cancer [118]. Compared with other treatment methods such as radiotherapy and chemotherapy, tumor vaccines offer the advantages of high specificity and a low incidence of adverse effects. However, owing to current technological limitations, responses to therapeutic tumor vaccines remain unsatisfactory.

### Adagloxad simolenin

Adagloxad simolenin is an active anti-cancer immunogen, which targets the Globo-H antigen which is highly prevalent on the surface of various cancers including TNBC [119]. It can be used as a target to identify cancer cells (which may stimulate the human immune response) and eliminate malignant tumors. On combined with the saponin adjuvant, OBI-821, adagloxad simolenin can induce an immune response to challenge tumor cells. An ongoing international multicenter phase III study (NCT03562637) is evaluating the anti-Globo-H vaccine in high-risk early Globo H-positive TNBC [120].

### α- lactalbumin engineered vaccine

The α-lactalbumin is being considered a potential target for developing a vaccine to treat and prevent TNBC. Although this protein does not exist in normal and aging tissues after lactation, it exists in most TNBCs. Activating the immune system against this redundant protein may provide pre-emptive immune protection against α- lactalbumin protein expression. In addition, the vaccine contains an adjuvant that can stimulate the innate immune response, thereby promoting the production of a specific immune response against tumors and preventing their further progression. In a study performed in a mouse model, α- lactalbumin engineered vaccine boosts antitumor immunity in breast cancer which could prevent breast cancer recurrence [121]. An open-label phase I clinical trial (NCT04674306)is ongoing to determine the safety as well as the optimal dose of α- lactalbumin vaccine to treat TNBC patients with a high risk of recurrence.

### DR5 DNA vaccine

DR5, also referred to as TNF-related apoptosis-inducing ligand receptor 2, has the ability to attach to TNF-related apoptosis-inducing ligands and facilitate cellular apoptosis. In a study using the BALB/c mouse model, a DR5 DNA vaccine induced the expression of proapoptotic antibodies, thereby triggering tumor cell apoptosis. In addition, the vaccine-induced DR5-specific T cells to secrete IFN-γ. The investigators concluded that DR5 can be used as an immunogenic target for a TNBC vaccine [122].

### DC fusion vaccine

A study evaluated a DC fusion vaccine in TNBC. Compared with the control group, the group that was administered a co-culture of DC, T cells, and TNBC cells (created by electrofusion technology) demonstrated significant stimulation of T cell expansion. The increased levels of IL-12 and IFN-γ demonstrated the specific cytotoxic effect of the DC fusion vaccine on tumor cells [123].

### Personalized peptide vaccine

Research has indicated the efficacy of personalized peptide vaccination (PPV) in managing TNBC [124]. During a phase II study involving patients with metastatic and recurrent TNBC, 1/18 patients each developed a complete and partial immune response after PPV administration (NCT02427581). PPV appeared to be safe, as it did not lead to any serious adverse effects in these patients. These results reveal that PPV has a potential effect in treating patients with TNBC [125].

## Cellular immunotherapy

### Chimeric antigen receptor T cell therapy

Chimeric antigen receptor T cell (CAR-T) therapy has shown excellent therapeutic effects or potential in treating diverse forms of cancer. Although breakthroughs have been achieved in hematological tumors, their use in the field of solid tumor therapy remains in the explorative stage. Studies in patients with TNBC have identified an antigen target related to breast CSCs (disialoganglioside GD2), which is widely found in highly invasive breast cancer subtypes; GD2-CAR-T cells were therefore created. In a study on a mouse model, GD2-CAR-T cells demonstrated a significantly higher inhibitory effect on tumor cell growth compared to CD19-CAR-T cells. Notably, no metastatic cancer cells were observed in the lungs of mice treated with GD2-CAR-T cells, all mice treated with CD19-CAR-T cells exhibited metastatic cancer cells in the lung [126]. Investigators have used TAB004 (a highly specific monoclonal antibody against the tumor-associated form of MUC1) to engineer the MUC28z (a chimeric antigen receptor) fusion molecule and generate CAR T cells; they also performed phenotypic and functional analyses. The results showed that MUC28z CAR-T cells can efficiently suppress the proliferation of TNBC tumors, under both in-vivo and in-vitro conditions. The CAR-T cells exhibited significant therapeutic promise in combating MUC1-positive TNBC tumors [127]. In a study on a TNBC mouse model, a fibrin gel containing CAR-T cells was applied to the surgical wound after partial tumor resection. The CAR-T cells significantly eliminated residual tumor cells, allowing the mice to survive. The results indicate that CAR-T cells could potentially act as a supplementary approach to enhance the efficiency and success rate of surgery in TNBC [128].

### NK cell therapy

The regulation of NK cell activities, such as degranulation, cytokine secretion, and cytotoxicity, is determined by the combination of signals received from activating and inhibitory receptors. NK cells possess the ability to identify and eliminate tumor cells that exhibit diminished or suppressed expression of MHC-I, a phenomenon referred to as the “missing self” recognition mechanism [129]. NK cells possess the ability to not only secrete cytotoxic particles, such as perforin and granase, via exocytosis but also directly induce the lysis of target cells and initiate cell apoptosis by activating the expressionof FASL/TRAIL in NK cells. Furthermore, other immune cells can be recruited to generate secondary immune responses by synthesizing and secreting IFN-γ, TNF-α, and chemokines [130, 131]. Additionally, another significant mechanism employed by NK cells in tumor eradication is antibody-dependent cell-mediated cytotoxicity (ADCC), which triggers the degranulation response of NK cells to eliminate target cells that are coated with antibodies. Presently, the repertoire of NK cell approaches employed in tumor mmunotherapy encompasses the following: in vitro administration of activated autologous or allogeneic NK cells; the induction of antibody-specific cytotoxicity through the amalgamation of NK cells and monoclonal agents (e.g., ICIs); and the implementation of CAR-NK cell immunotherapy. Notably, NK cells derived from cancer patients exhibit comparable expansion potential to those derived from healthy donors, exhibit cytotoxicity against TNBC cell lines and xenograft tumor models, and can be harmoniously integrated with existing.

cancer treatments [132]. To enhance the functionality of NK cells in patients with TNBC, it has been observed that the combined approach of radiotherapy (RT) and NK cell therapy can effectively facilitate the infiltration and migration of NK cells into the tumor lesion within the xenograft tumor model of TNBC. Consequently, this approach leads to the reduction of tumor burden and tumor growth. Notably, compared to the NK group, the RT + NK group demonstrated a significantly prolonged presence of NK cells within the primary tumor tissue. Furthermore, the combined therapy of RT + NK cells effectively suppressed the dissemination and distant metastasis of breast cancer cells to the lymph nodes, specifically in the lung and liver [133]. Avelumab, a checkpoint inhibitor targeting PD-L1, demonstrated the ability to elicit cytotoxic effects mediated by NK cells in TNBC patients [134]. Chen et al. developed a nano-system incorporating selenium, which effectively increased NKG2D expression on NK cells and NKG2DLs expression on tumor cells. This augmentation enhanced the recognition and cytotoxicity of NK cells towards tumor cells, thereby bolstering the immune response against tumors facilitated by NK cells [135]. Additionally, studies have shown that Ruthenium complexes and Aptamer-engineered approaches can enhance the sensitivity of TNBC to NK cell therapy and modulate the immune microenvironment [136, 137]. The presence of an immature NK cell population (CD11b CD27 Socs3high) in TNBC has been found to diminish granules-mediated cytolysis and enhance the expression of Wnt16 ligand, which has been implicated in tumor progression and metastasis [138]. The promotion of TNBC progression can be facilitated by the depletion of NK cells, inhibition of Wnt secretion, utilization of immunotherapy involving anti-PD-L1 antibodies, or a combination of chemotherapy and immunotherapy [139].

CAR-NK is an application of genetic engineering cellular therapy that incorporates a chimeric antibody into NK cells, which can significantly improve the specificity of NK cell’ s therapeutic effects. Notably, a study demonstrated that EGFR-CAR NK cells exhibited cytotoxicity and anti-tumor properties against TNBC cell lines with high EGFR expression.

Optimizing the cytotoxic potential of NK cells appears to heavily rely on the tailored design of CAR structures dedicated to NK cells [140]. In addition, it is imperative to optimize the procedures for amplifying and activating harvested NK cells to obtain a large number of memory-like, unexhausted, and homogeneous populations of NK cells in clinical practice. Moreover, the effectiveness of CAR-NK cells in TNBC treatment might necessitate additional modifications to enhance the trafficking capabilities and reduce the sensitivity to immunosuppression in the TME.

### Cytokine-induced killer cell therapy

Cytokine-Induced Killer (CIK) cells are a diverse group of polyclonal T lymphocytes acquired by expanding lymphocytes in vitro, and exhibit functional and phenotype characteristics of both T cells and NK cells. These cells express two membrane protein molecules, CD3 and CD56, simultaneously, earning them the designation of NK cell-like T lymphocytes. Due to their potent tumor cell recognition abilities, CIK cells are often likened to “cell missiles” that can accurately target tumor cells without harming normal cells. In the case of patients with TNBC, repeated administration of CIK cells and cetuximab to patients with TNBC has been shown to significantly impede the growth, metastasis, and dissemination of TNBC cells and xenotransplanted tumors in lymph nodes and lungs [141]. The use of the treatment approach successfully inhibits the reoccurrence and extends the lifespan of patients with TNBC who have lymph node spread, an advanced TNM stage, and poor histological grade [142].

**Fig. 5:**
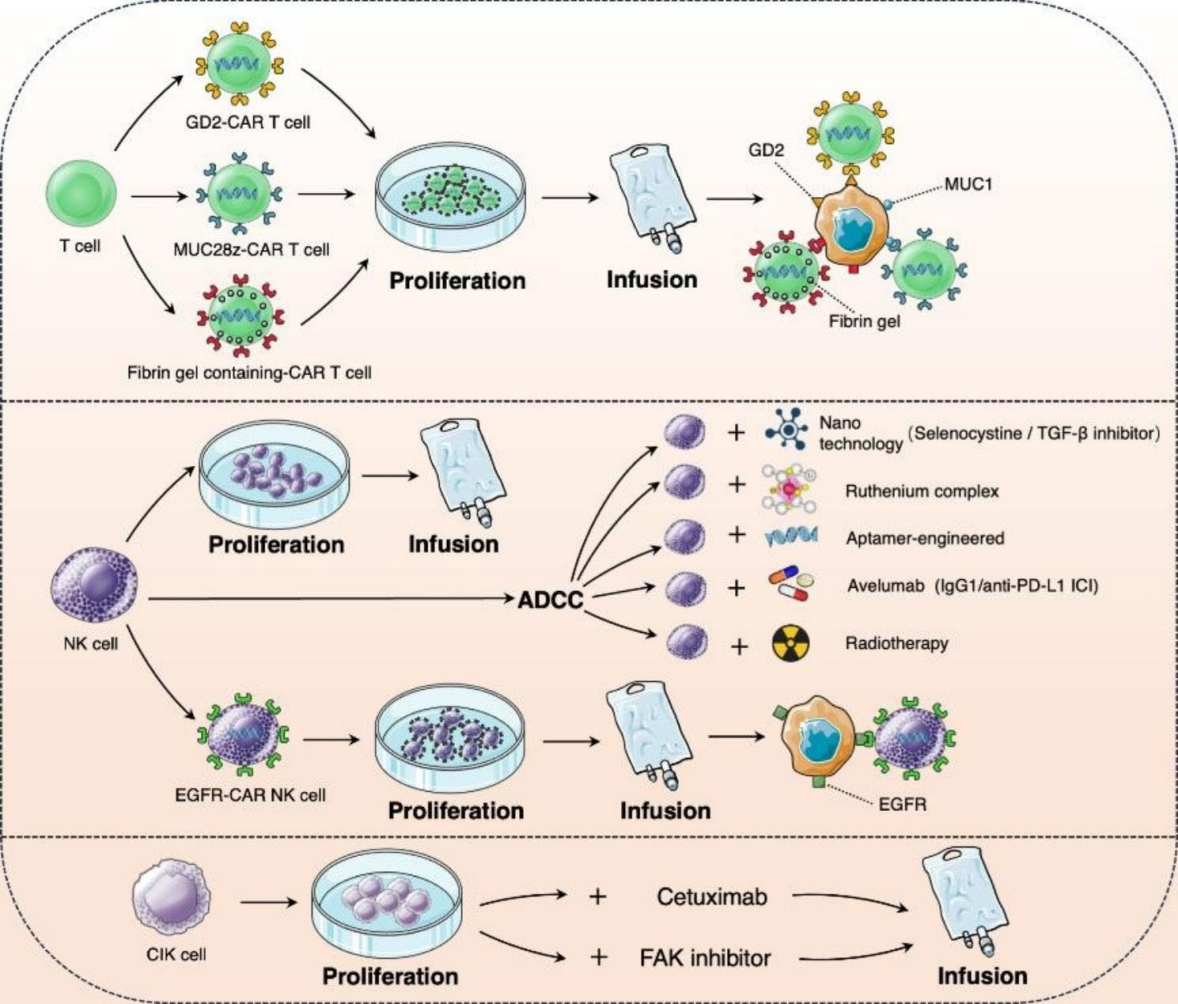
The diagram CAR-T cell, NK cell and CIK cell separately or with others anticancer approaches to treat TNBC. GD2-CAR-T cells, MUC28z CAR-T cells and a fibrin gel containing CAR-T cells demonstrate a significantly higher inhibitory effect on tumor cell growth in TNBC. NK cells derived from cancer patients exhibit heightened cytotoxicity against TNBC cell lines and xenograft tumor models. The combination therapy of RTand NK cells, Avelumab, Selenocystine/TGF-β Inhibitor, Ruthenium complexes, Aptamer-engineered approach enhance the cytotoxic effects mediated by NK cells. EGFR-CAR NK cells exhibited cytotoxicity and anti-tumor properties against TNBC cell lines. The combination administration of CIK cells and cetuximab or CIK cells and FAK inhibitor has been shown to significantly impede the growth, metastasis, and dissemination of TNBC

CIK cells have demonstrated potential benefits in patients with various tumor types, however, their effectiveness varies among individuals. The role of Focal adhesion kinase (FAK).

in controlling cell invasion and migration is vital, which in turn affects the vulnerability of tumor cells to CIK cells. Current evidence suggests a favorable connection between PD-L1 and FAK mRNA expression in PD-L1 positive TNBC, indicating a possible link.

between FAK and the function of immune checkpoints. Furthermore, studies have shown.

that the anti-PD-L1 antibody atezolizumab greatly augments the suppressive impact of FAK inhibitors on cancer cells [143]. The application of FAK inhibitors in the treatment of TNBC cells, along with co-culturing with CIK cells, resulted in a higher incidence of cell death, suggesting that FAK enhances the susceptibility of tumor cells to CIK cells [144]. A study conducted in vivo has substantiated that the combined administration of FAK inhibitors and CIK cells surpasses the efficacy of either treatment in inhibiting tumor growth [145]. Consequently, the integration of CIK cell therapy with PD-L1 inhibitors and FAK inhibitors holds promise as a potentially efficacious and innovative treatment approach for patients with TNBC. The immunotherapy of CAR-T cell, NK cell and CIK cell separately or with others anticancer approaches to treat TNBC is shown in Fig. 5.

## Bispecific antibody approaches for TNBC therapy

**Fig. 6:**
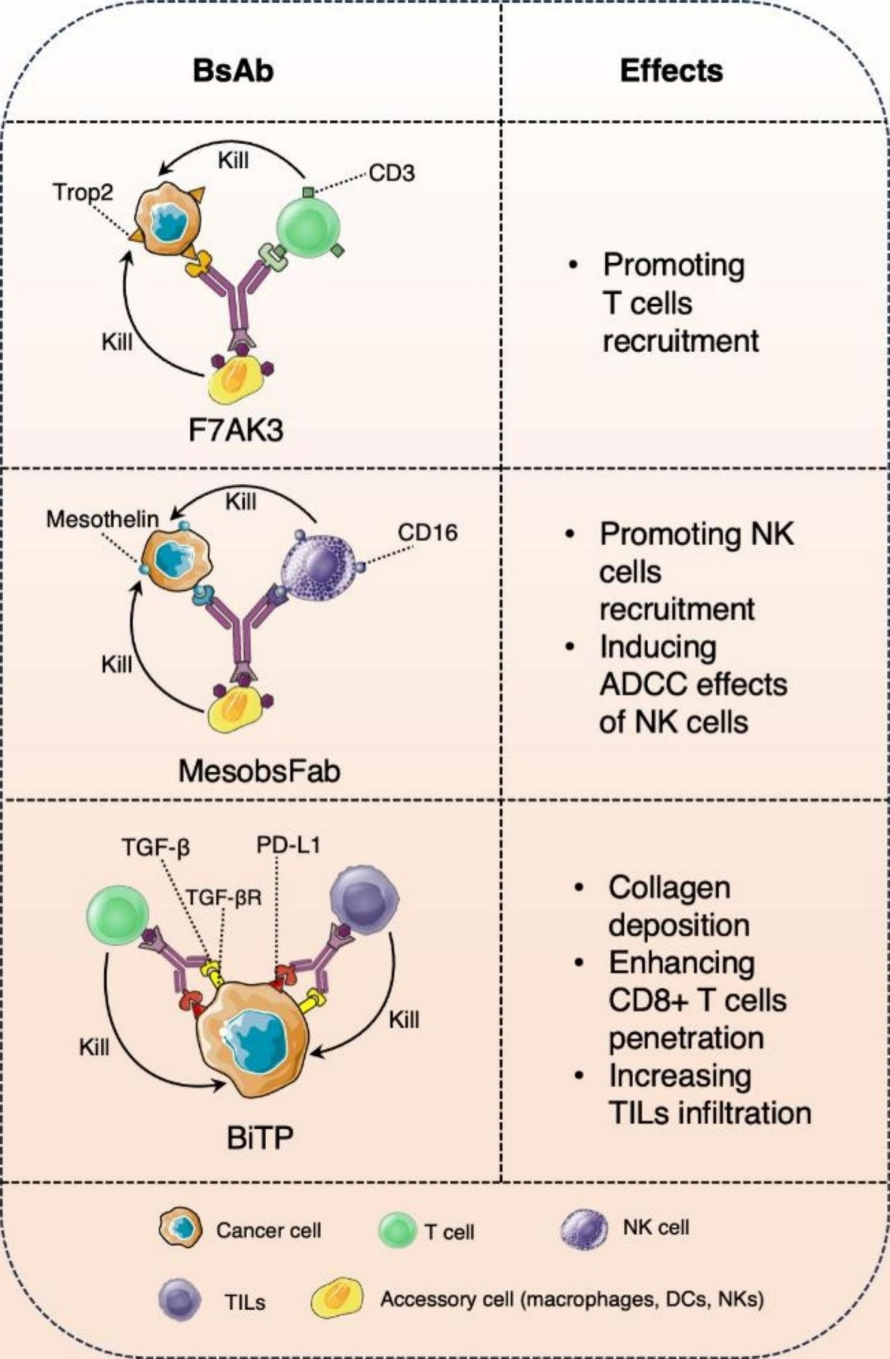
The diagram of bsAb types applied to treat TNBC. F7AK3 with cell surface antigen TROP2 and CD3 facilitates the recruitment of T cells. MesobsFab elicits the recruitment and infiltration of NK cells and leads to the induction of ADCC effect in mesothelin-positive tumors. BiTP was developed for TGF-β and human PD-L1 by using the Check-BODY platform, which increase the collagen deposition, TILs infiltration, CD8 + T cells penetrating

Bispecific antibody (BsAb) refers to an artificially engineered antibody that can specifically bind two antigens or antigenic epitopes simultaneously, serving as a bridge connecting two antigens (epitopes). This antibody does not occur naturally but is synthesized through cell fusion or recombinant DNA techniques. The main mechanism of BsAb in anti-tumor therapy involves (1) The recruitment of T cells or NK cells and augmenting cytotoxicity effect on tumor cells (2) Simultaneously blocking two different signaling pathways to synergistically inhibit the growth and proliferation of tumor cells (3) Concurrently targeting different antigens or epitopes on the cell surface to enhance the specific binding with tumor cells. F7AK3 is a BsAb that has been developed and can specifically bind to the trophoblast cell surface antigen 2 (TROP2) and CD3 of TNBC cell lines and primary tumor cells, thereby recruiting T cells. A study found that F7AK3 inhibits the growth of a xenograft NCG immunodeficient mouse model in TNBC [146]. Consequently, the potential utilization of F7AK3 alone or in combination with ICIs for immunotherapy in advanced TNBC necessitates further investigation through clinical trials. MesobsFab, a Fab-like format, targeting mesothelin and CD16, which facilitates the recruitment and infiltration of NK cells into tumor spheres, leading to strong, dose-dependent cell-mediated cytotoxicity in mesothelin-positive TNBC [147]. BiTP, a kind of BsAb, was developed for TGF-β and human PD-L1 using the Check-BODY platform. The administration of BiTP in the humanized TNBC model resulted in the elevated infiltration of TILs, CD8 + T cells, and NK cells, thereby reshaping the TME [148]. The structure and effect of F7AK3, MesobsFab, and BiTP was illustrated in Fig. 6. Furthermore, numerous therapeutic targets for TNBC have been integrated into BsAb constructs, including CEACAM5, EphA10, P-cadherin, EpCAM, and EGFR [149–154]. Recently, there has been an evaluation of BsAb that specifically target receptors, such as EGFR, HER3, and Notch on TNBC cells [147, 155–159]. The utilization of BsAb for the treatment of TNBC is experiencing a persistent surge in momentum. Numerous targets specific to TNBC have emerged as potential candidates for the future advancement of BsAb [159].

Currently, diverse technologies for BsAbs production exist, which can be broadly categorized into two groups: BsAbs with the Fc domain and BsAbs without the Fc domain. The inclusion of the Fc domain in BsAbs serves to enhance stability and prolong their half-life. Nevertheless, the interaction between the Fc domain and its receptors or complements may trigger ADCC, resulting in non-specific immune responses. On the other hand, Bispecific T cell engagers (BiTEs) are a type of BsAb that lack the Fc domain, thereby circumventing cytotoxicity. However, BiTEs exhibit a short half-life in the bloodstream, necessitating continuous infusion. One arm of the BiTE molecule specifically binds to the CD3 antigen on the surface of T cells, while the other arm binds to the tumor-associated antigen (TAA). Upon binding to their respective targets, the formation of synapses between T cells and cancer cells occurs, leading to the release of perforin and granzyme [160], which ultimately results in the death of cancer cells. In comparison to conventional antibodies, BiTE exhibits higher tissue permeability, increased efficiency in killing tumor cells, requires lower dosages, and produces stronger therapeutic effects [161]. However, the full extent of the benefits of this combination therapy in TNBC remains unclear due to the inherent heterogeneity, necessitating further investigation.

The utilization of BsAb has emerged as a significant and promising constituent of the forthcoming therapeutic antibody generation, owing to its capacity to effectively target two epitopes within tumor cells or the TME. Presently, numerous preclinical and clinical trials, including NCT05403554, NCT03219268, and NCT04424641, among others, are either in progress or have been concluded. Nevertheless, the utilization of BsAb therapeutics in the context of tumor treatment encounters significant hurdles, encompassing tumor heterogeneity and mutation burden, the imperative for repeated administration, deleterious adverse reactions, and off-target effects.

In this summary, we have provided an overview of the fundamental and clinical investigations conducted on immunotherapy in TNBC (Tables 1 and 2). Moreover, the scope of immunotherapy application in TNBC is expected to expand, encompassing the entire domain of breast cancer, ultimately benefiting a substantial proportion of patients.

**Table 1:** Clinical trials of immunotherapy in TNBC

Trial number	Treatment	Subject	Results in patients with TNBC	Refs
NCT02447003	Pembrolizumab	Metastatic TNBC	ORR in ITT: 5.3%; ORR in patients with PD-L1+: 5.7%; mPFSb : 2.0 m; mOSb : 9.0 m	[54]
KEYNOTE-012	Pembrolizumab	Metastatic TNBC	ORR: 18.5%; mPFS: 1.9 m; mOS: 11.2 m	[53]
NCT01375842	Atezolizumab	Metastatic TNBC	ORR in ITT: 10.0%; ORR in patients with PD-L1+: 12.0%; mPFSb: 1.4 m; mOSb : 8.9 m	[58]
NCT01772004	Avelumab	Metastatic TNBC	ORR in ITT: 5.2%; ORR in patients with PD-L1+: 44.0%; mPFSb : 1.4 m; mOSb : 9.2 m	[59]
NCT02838823	JS001	Metastatic TNBC	ORR in ITT: 5.0%; ORR in patients with PD-L1+: 11.1%; ORR in patients with PD-L1– tumours: 0%; mPFSb : 1.8 m	[57]
NCT02447003	Pembrolizumab	Metastatic TNBC	ORR: 21.4%; mPFS: 2.1 mo; mOS: 18 m	[162]
	Durvalumab + Tremelimumab	Metastatic TNBC	Induce plasma cells to produce specific antibodies, Activated and Increased T cell	[66]
NCT02657889	Pembrolizumab + Niraparib	Advanced/metastatic TNBC	ORR 21%, DCR 49%	[94]
NCT03330405	ICIs + Avelumab + Talazoparib	Advanced TNBC	ORR = 18.2%	[95]
NCT0265788	Niraparib + Pembrolizumab	Advanced/Metastatic TNBC	ORR in ITT: 29.0%; ORR in patients with PD-L1 + tumors 32%	[94]
NCT03394287	Camrelizumab + VEGFR2 inhibitors	Advanced TNBC	shown good efficacy in patients with PD-L1 negative tumors or those receiving advanced lines of chemotherapy	[100]
NCT02555657	Pembrolizumab vs. TPCe	Metastatic TNBC	ORR in patients with CPS ≥ 10: 17.7% vs. 9.2%; mPFS in patients with CPS ≥ 10: 2.1 mo vs. 3.4 m; mOS in patients with CPS ≥ 10: 12.7 m vs. 11.6 m (P = 0.06)	[163]
NCT03310957	Ladiratuzumab vedotin + Pembrolizumab	Metastatic TNBC	ORR: 54.0%	[164]
NCT03742102	Durvalumab + Trastuzumab deruxtecan	Metastatic TNBC	ORR: 66.7%	[165]
NCT02513472	Eribulin + Pembrolizumab	Metastatic TNBC	ORR overall: 23.4%; ORR in patients with PD-L1 + tumors, first-line setting: 34.5%;	[166]
NCT02734004	Olaparib + Durvalumab	Metastatic TNBC	ORR: 58.8%; mPFS: 4.9 m; mOS: 20.5 m	[96]
NCT03752723	GX-I7 + Pembrolizumab	Metastatic TNBC	ORR: 13.3%	[167]
NCT02708680	Entinostat + Atezolizumab	Metastatic TNBC	ORR: 10.0%; mPFS: 1.68 m; mOS: 9.4 m	[107]
NCT03797326	Lenvatinib + Pembrolizumab	Metastatic TNBC	ORR: 29.0%	[168]
NCT02819518	TPCg + Pembrolizumab/Placebo	Metastatic TNBC	ORR (co-primary end point) in patients with CPS > 10: 53.2% vs. 39.8%	[81, 169]
NCT01042379	Paclitaxel with or without Pembrolizumab + adjuvant chemotherapy	Early-stage TNBC	pCR rate: 60% vs. 22%	[170]
NCT02685059	Nab-paclitaxel + Durvalumab/Placebo +Endocrine therapy +Durvalumab/Placebo	Early-stage TNBC	pCR rate in ITT: 53.4% vs. 44.2% (P = 0.29); pCR rate in patients with PD-L1 + on IC: 58.0% vs. 50.7%	[75, 76]
NCT02622074	Pembrolizumab + Anthracycline + Taxane-based Chemotherapy with or without Carboplatin + Adjuvant chemotherapyc	Early-stage TNBC	pCR rate overall: 60%; pCR rate in patients with CPS > 1: 64%; pCR rate in patients with CPS > 30: 72%	[72]
NCT03197935	Nab-paclitaxel + Atezolizumab /Placebo + Adjuvant chemotherapy+ Atezolizumab/Placebo	Early-stage TNBC	pCR rate in ITT: 58% vs. 41% (P = 0.004); pCR rate in patients with PD-L1 + on IC: 69% vs. 49% (P = 0.02);	[78]
NCT03036488	Anthracycline, taxane and carboplatin-based chemotherapy + Pembrolizumab/Placebo + Adjuvant Chemotherapy/Endocrine therapy	Early-stage TNBC	pCR rate: 63.0% vs. 55.6%	[73]
NCT02620280	Nab-paclitaxel + Carboplatin with or without Atezolizumab	Early-stage TNBC	pCR rate in ITT: 43.5% vs. 40.8%; pCR rate in patients with PD-L1 + on IC: 51.9% vs. 48.0%	[77]
NCT02819518	Pembrolizumab + Chemotherapy	Advanced TNBC	mOS was higher than placebo-chemotherapy group	[80]
NCT01633970	Nab-paclitaxel +Atezolizumab	Metastatic TNBC	ORR in ITT: 39.4%; mPFSb : 5.5 m; mOSb : 14.7 m	[171]
NCT03800836	Ipatasertib + Atezolizumab + Paclitaxel/ Nab-paclitaxel	Metastatic TNBC	ORR in ITT: 73.0%; ORR in patients with PD-L1 + tumors: 82%	[105]
NCT04129996	Angiogenesis inhibitor + Camrelizumab + Chemotherapy	Advanced immunomodulatory TNBC patients	objective response rate was 81.3%, mPFS was 13.6 m, PFS was higher in CD8 + than in CD8- patients	[101]
NCT02322814	Cobimetinib + Atezolizumab +Paclitaxel/Nab-paclitaxel	Metastatic TNBC	ORR overall: 31.7%; ORR in patients with PD-L1 + tumors: 44% with paclitaxel, 33% with nab-paclitaxel	[108]
NCT02425891	Nab-paclitaxel + Atezolizumab/Placebo	Metastatic TNBC	ORR in ITT: 56.0% vs. 45.9% (P = 0.002); ORR in patients with PD-L1 + tumors: 58.9% vs. 42.6% (P = 0.002)	[172]
NCT03125902	Paclitaxel + Atezolizumab/Placebo	Metastatic TNBC	ORR in ITT: 53.6 vs. 47.5; ORR in patients with PD-L1 + tumors: 63.4% vs. 55.4%	[173]
NCT02299999	Durvalumab vs. Chemotherapy	Metastatic TNBC	mOS in all patients with TNBC: 21.2 m vs. 14 m (P = 0.04); mOS in patients with PD-L1 + tumors: 27.3 m vs. 12.1 m (P = 0.07)	[174]
NCT04674306	α- lactalbumin engineered vaccine	TNBC	Ongoing	[121]
NCT03562637	Adagloxad Simolenin(OBI-822/OBI-821)	High risk early Globo-H positive TNBC	Longer PFS in vaccinated patients who developed anti-Globo H (anti-GH) IgG	[120]
NCT02427581	Personalized peptide vaccine	Metastatic and recurrent TNBC	1/18 patients developed a complete and partial immune response	[125]
NCT01395056	cytokine-induced killer cell (CIK) infusion + chemotherapy	Post-mastectomy TNBC/TNBC	Prevented disease recurrence and prolong survival,	[142]

**Table 2:** Immunotherapy methods and mechanisms of TNBC in vivo and in vitro

Treatment	Treatment mechanism	in vitro or in vivo	Result	Refs
Rg3 liposome + Docetaxel	Inhibited TGF-β secretion, reduced CAFs and collagens	4T1 mice model	improve the anti-tumor effect of DTX	[24]
Anacardic acid(6SA)	Activate immune cells, augment the secretion of IFN-γ and TNF-α, and improve TME	4T1 mice model	Induced cell apoptosis, reduced tumor volume	[27]
CpG-oligodeoxynucleotides	Combined with anti PD-1 inhibitors, stimulated plasmacytoid DCs to produce IFN α and β	TNBC mice	Upregulated IFN release; increase antigen-specific CD8 + T cell infiltration	[37]
Radiotherapy + ICIs	Upregulated the expression of genes containing immunogenic mutations, induced CD8 + and CD4 + T cells	Low immunogenicity TNBC mice	Enhanced anti-tumor efficacy	[84]
Radiotherapy + anti-PD-1 antibody	Reduced tumor cell growth not only in the irradiated site but also in non-irradiated sites	Poorly immunogenic metastatic 4T1 mice	Reduced tumor cell growth, improved survival rates, and inhibited lung metastasis	[85]
AdLyp.sT and mHAdLyp.sT	Expressed sTGFβRIIFc that inhibit TGFβ pathways, enhanced the efficacy of anti- CTLA-4 and anti-PD-1	4T1 mice model	Inhibited tumor growth and metastases, augmented anti-PD-1 and anti-CTLA-4 therapy	[89]
Albumin nanoparticle	Contain PI3K-γ inhibitor eganelisib (IPI-549) and PTX	TNBC mice	Enhanced the efficacy of α-PD1 with longer PFS and a better remission rate	[104]
2 F-Fuc + anti-PD-L1	Enhance the activation of T cells, blocked B7H3 core focusing	TNBC mice	Significantly inhibited tumor growth	[106]
ICD + PD-1/PD-L1 blockade + iPPO + MW	Facilitated the maturation of DCs and reversed the immunosuppressive environment	4T1 mice model	Amplified the systemic anti-tumor immune response	[114]
mEHGZ + anti-PD-L1	Induced IFN-γ and CD8 + T cell infiltration	4T1 mice model	Enhanced the sensitivity and efficacy of ICIs	[115]
Nanoparticles (NPs)	Delivered an mRNA vaccine encoding tumor antigen MUC1 to DCs to activate and expand tumor-specific T cells	4T1 mice model	NPs improved the stability, sustainability, and expression level of messenger RNA-based vaccines	[117]
BiTP (Anti-TGF-β/PD-L1 bispecific antibody)	Effectively counteracted TGF-β-Smad, PD-L1, PD-1 penetration, increased TIL NFAT signaling, decreased collagen deposition, enhanced CD8 T cell	Cell, TNBC mice models	Superior antitumor activity relative to anti-PD-L1 and anti-TGF-β monotherapy	[175]
α-lactalbumin engineered vaccine	α- lactalbumin specific T cells induce tumor inflammation and cytotoxicity	4T1 mice model	Boosted antitumor immunity, provide significant protection and therapy against growth of autochthonous tumors in MMTV-neu and MMTV-PyVT transgenic mice and against 4T1 transplantable tumors	[121]
TMAO	Remodel of the TME	4T1 mice model/cell	Enhanced the infiltration and killing function of CD8 + T cells, induce tumor cell pyrosis	[29]
Rg3 + paclitaxel	Inhibit NF-κB activation, decreased NF-κB p65 and Bcl-2 protein expressions, increased Bax and Caspase-3 protein expressions	MDA-MB-231 cell	Rg3 promoted cytotoxicity and apoptosis of Paclitaxel	[25]
Pos3Aa-p53	Enhance the sensitivity to the chemotherapy drug 5-fluorouracil	TNBC mice, MDA-MB-231、549、4T1 cell	Resulted in the restoration of p53 function in p53-deficient cancer cells, and sensitized them to 5-fluorouracil chemotherapy	[42]
AZD5069 + Atezolizumab	Reduce chemoresistance	MDA-MB-231 cell	Enhanced the efficacy of anti-PD-L1 therapy	[45]
PmTriTNE@CDA	Upregulate the expression of type I IFNs	4T1 and MDA-MB-231 cell	Improved immunogenicity	[46]
Pos3Aa-p53 +anti-PD-1 antibodies	Induce anti-tumor immune memory	Mouse 4T1 breast cancer cells	Enhanced clinical response to anti-PD-1 therapy	[43]
Ad.sTbetaRFc	High levels of viral replication	MDA-MB-231cell	More than 85% of the tumors showed reduction	[87]
DR5 DNA vaccine	induced DR5-specific T cells to secrete IFN-γ	BALB/c mouse model	Triggered tumor cell apoptosis	[122]
DC fusion vaccine	increased levels of IL-12 and IFN-γ	4T1 mice model, Mouse 4T1 breast cancer cells	Had specific cytotoxic effect on tumor cells	[123]
Chimeric antigen receptor T cell therapy	delivery of CAR T cells in fibrin gel applied into the resected tumor cavity	TNBC mouse model, MDA-MB-231	Significantly eliminated residual tumor cells in mice TNBC models	[128]
MUC28z CAR-T cells	Increased production of Granzyme B, IFN-γ and other Th1 type cytokines and chemokines	TNBC cell, breast cancer mouse model	Inhibited the growth of TNBC tumors in-vivo and in-vitro conditions	[127]
Ruthenium complexes + Aptamer-engineered approaches	Induce robust ROS generation, activate multiple apoptosis-related receptors, enhance the sensitivity of TNBC to NK cell therapy	MDA-MB-231 cells	Modulated the immune microenvironment	[136]
Repeated administration of CIK cells and cetuximab	Foster potent antibody-dependent cell–mediated cytotoxicity (ADCC)	MDA-MB-231 cells, TNBC mouse model	Significantly impede the growth, metastasis, and dissemination of TNBC cells and xenotransplanted tumors in lymph nodes and lung	[176]
Atezolizumab +FAK inhibitors	Potentiates T cell-mediated cytotoxicity and significantly enhance the inhibitory effects of FAK inhibitors	TNBC cells	Suppressed TNBC cell invasion and motility	[143]
FAK inhibitor + CIK cells	FAK suppression promote cytotoxicity induced by CIK cells	MDA-MB-231 cell, TNBC mouse model	Significantly suppressed tumor growth than the treatment of FAK inhibitor or CIK cells alone	[145]
Anti-TROP2xCD3 bispecific antibody F7AK3	F7AK3 recruits T cells to TROP2 tumor cells	TNBC cells, Mouse TNBC xenograft model	Inhibited the TNBC tumor growth	[146]
MesobsFab	facilitates the recruitment and infiltration of NK cells into tumor sphere	TNBC cell, mouse model	Potent dose-dependent cell-mediated cytotoxicity	[177]

## Discussion

The IMpassion130 study was the first to indicate the value of immunotherapy in the management of TNBC. Notably, TNBC is the most studied malignant tumor in the field of immunotherapy. Although immunotherapy offers better safety and tolerability and a longer median PFS and OS, many issues remain to be resolved. Firstly, effective immune markers for excluding TNBC immune-dominant populations are lacking. Studies categorizing high, medium, and low-risk populations based on marker threshold values (and thereby demonstrating the mechanism of immunotherapy) are also lacking. Secondly, immunotherapy remains at an early stage of development, and not all immune checkpoints that may be relevant to patients with TNBC have been identified. The ICIs mentioned in this review are unlikely to apply to all patients with TNBC. In order to identify the recipients of precise treatment, it is imperative to discover more precise predictive biomarkers. Additionally, the reports from the literature show that immunotherapy is associated with risks of developing irreversible endocrine disorders including hypoglycemia, hyperthyroidism, type I diabetes, adrenal insufficiency, and hypophysitis. it is crucial to prioritize the management of adverse reactions in clinical trials. Should OS and DFS be considered alternative endpoints for neoadjuvant immunotherapy in TNBC? In other types of solid tumors, the true advantages of immunotherapy are often observed through the extension of OS. Consequently, pCR may not be the most optimal alternative endpoint for the neoadjuvant treatment phase. Therefore, it is highly recommended to investigate appropriate research endpoints in future clinical studies on neoadjuvant immunotherapy in TNBC. In order to enhance the efficacy of immunotherapy for TNBC, it is imperative to explore a more potent combination therapy that can effectively eradicate tumors, augment tumor antigen exposure, facilitate antigen presentation, and prevent immune evasion.

## Prospect

The combination with other treatment modalities currently offers an effective approach for immunotherapy in TNBC. The enhancement of immunotherapy response rates can be achieved through the integration of various treatment strategies, encompassing combination chemotherapy, targeted therapy, alternative immunotherapy approaches, cellular therapy, radiotherapy, etc. Future advancements in the field lie within the realm of immunosuppressive combined chemotherapy/targeting/novel immunotherapies, including inhibitors targeting BRCA/PI3K/AKT/mTOR/MEK/CDK4/6, androgen receptor inhibitors, ADC drugs, tumor vaccines, oncolytic viruses, and adoptive immunotherapies such as TIL and CAR-T. Future clinical trials need to consider the use of traditional therapies, ICIs, and combination therapies based on the specific molecular subtypes of TNBC, the number of TILs, and PD-L1 expression. It is also necessary to identify patients who may safely receive immunotherapy and even benefit from different combination therapies.

## Conclusion

Although studies have explored the molecular characteristics of TNBC and their dynamic relationship with the TME and immune cells in detail, the considerable heterogeneity in this entity warrants further studies to understand its molecular characteristics and microenvironmental structure. Existing multi-dimensional omics data need to be integrated to ensure a more accurate classification of TNBC subtypes. New targets are being continually developed to achieve good efficacy and improve individualized precision treatment approaches. Their clinical application is expected to confer better therapeutic outcomes in TNBC patients.

## Data Availability

Not applicable.

## Abbreviations

2 F-Fuc: 2-fluoro-L-focus
ACSL3: Acyl-CoA Synthetase Long Chain Family Member 3
ADCC: Antibody-dependent cell-mediated cytotoxicity
ADV/HSV-tk: Adenovirus mediated herpes simplex virus thymidine kinase
APCs: Antigen presenting cells
B7H3: B7 homologous 3
BiTEs: Bispecific T cell engagers
BsAb: Bispecific antibody
CAFs: Cancer-associated fibroblasts
CAR-T: Chimeric antigen receptor T cell
CDA: C-di-AMP
CIK: Cytokine-induced killer
CpG-ODN: CpG oligodeoxynucleotides
CpG: Cytosine-phosphorothioate-guanine
CPS: Comprehensive positive score
CSCs: Tumor stem cells
CTLA-4: Cytotoxic T-lymphocyte associated antigen 4
CXCR2: Chemokine receptor 2
DCR: Disease control rate
DCs: Dendritic cells
ECM: Extracellular matrix
EPI: Epirubicin
ERK1/2: Extracellular signal-regulated kinase 1/2
FAK: Focal adhesion kinase
HSV: Herpes simplex virus
ICD: Immunogenic cell death
ICIs: Immune checkpoint inhibitors
IFN-: γ interferon γ
IFNs: Interferon
IL-12: Interleukin-12
IM: Immunomodulatory
iPP: Polyacrylic acid polymer lactic acid and glycolic acid perfluoronaphthalane
iPPO: O3 loaded iPP particles
LAM: Lipid-associated macrophage
LCP: Lipid calcium phosphate
LOX: Lysyl oxidase
MAPK: Mitogen activated protein kinases
MES: Macrophage-enriched subtype
MHC-I: Major histocompatibility complex class I
mPEG: Methoxy polyethylene glycol
mTNBC: Metastatic triple negative breast cancer
MUC1: Mucin 1
MW: Microwave
NES: Neutrophil-enriched subtype
NK: NK
NO: Nitric oxide
OPN: Osteopontin
ORR: Objective response rate
OXPHOS: Oxidative phosphorylation
PARP: Poly ADP-ribose polymerase
PARPi: PARP inhibitors
PCR: Pathological complete response rate
PD-1: Programmed death receptor-1
PD-L1: Programmed death receptor-ligand 1
PFS: Progression-free survival
PI3K/AKT/mTOR: Phosphatidylinositol 3-kinase/protein kinase B/mammalian target of rapamycin
PPV: Personalized peptide vaccination
PPV: Personalized peptide vaccination
PTX: Paclitaxel
Rg3-DTX-LPs: Docetaxel loaded Rg3 liposome
RNA: Ribonucleic acid
ROS: Reactive oxygen species
SBRT: Stereotactic body radiotherapy
SHP2: SH2 protein tyrosine phosphatase 2
STING: Interferon gene stimulating factor
TAA: Tumor associated antigen
TAMs: Tumor-associated macrophages
TGF: β RIIFc TGF β Receptor II IgG Fc Fragment
TIL: Tumor infiltrating lymphocyte
TLR9: Toll-like receptor 9
TMAO: Trimethylamine oxide
TME: Tumor microenvironment
TNBC: Triple negative breast cancer
TNF-α: Tumor necrosis factor-α
Treg: Regulatory T cells
TriTNE: Nanotechnology × PD-L1/CD3 three specific T cell nano adapter
TROP2: Trophoblast cell surface antigen 2
VEGF: Vascular endothelial growth factor
