# Correction: Advances in immunotherapy for triple-negative breast cancer

**DOI:** 10.1186/s12943-023-01858-z

**Published:** 2023-09-14

**Authors:** Yang Liu, Yueting Hu, Jinqi Xue, Jingying Li, Jiang Yi, Jiawen Bu, Zhenyong Zhang, Peng Qiu, Xi Gu

**Affiliations:** 1 Department of Oncology, Shengjing Hospital of China Medical University, Shenyang, 110004 Liaoning Province China; 2 Department of Health Management, Shengjing Hospital of China Medical University, Shenyang, 110004 Liaoning Province China; 3 Department of Anesthesiology, Shengjing Hospital of China Medical University, Shenyang, 110004 Liaoning Province China

**Correction**: ***Mol Cancer *22, 145 (2023)**

10.1186/s12943-023-01850-7

Following the publication of the original article [1], the authors Yang Liu, Yueting Hu and Jinqi Xue are all the first author who contributed equally to the manuscript, however, the sentence ”Yang Liu, Yueting Hu and Jinqi Xue contributed equally to this work” is missing in the published version. The missing sentence has been added in this correction note article.
